# Differential Alteration of Gene Expression by Benzyl Adenine and *meta*-Topolin in In Vitro Apple Shoots

**DOI:** 10.3390/plants14233691

**Published:** 2025-12-04

**Authors:** Anita Király, Viktor Ambrus, Dóra Farkas, Neama Abdalla, Judit Dobránszki

**Affiliations:** 1 Centre for Agricultural Genomics and Biotechnology, University of Debrecen, 4400 Nyíregyháza, Hungary; gonda-kiraly.anita@agr.unideb.hu (A.K.); farkas.dora@agr.unideb.hu (D.F.); 2 Department of Biotechnology and Microbiology, Faculty of Science and Technology, University of Debrecen, 4032 Debrecen, Hungary; ambrus.viktor@science.unideb.hu; 3 Plant Biotechnology Department, Biotechnology Research Institute, National Research Centre, 33 El Buhouth St., Dokki, Giza 12622, Egypt; na.abdel-aal@nrc.sci.eg

**Keywords:** auxin transport, auxin signaling, cellular transport, catabolism, cytokinins, cv. Húsvéti rozmaring, *Malus x domestica* Borkh., phytohormones, redox homeostasis, transcription factors

## Abstract

Exogenous cytokinin supply is a crucial factor during the in vitro shoot multiplication of apples. *Meta*-topolin has been shown to cause improved multiplication rate, higher quality in vitro shoots with better rooting, and acclimatization ability than the widely used benzyl adenine. The effects of benzyl adenine and *meta*-topolin on mRNA transcription in in vitro shoots were analyzed by using mRNA-seq, bioinformatics analysis, GO annotation, and KEGG mapping. The present investigations revealed that there were about 6-fold more significantly up-, or down-regulated genes (DEGs) in shoots grown on the benzyl adenine-containing medium than in those grown on the *meta*-topolin-containing medium. DEG analyses showed that WRKYs, bHLH, and MYB were the most affected transcription factors after both cytokinin treatments, while the expression of MIKC-type MADS-box, ERF, and AP2 transcription factors changed only after benzyl adenine treatment. DEGs related to auxin transport and signaling, as well as auxin synthesis, were differently affected by the two cytokinins. The DEG encoding cytokinin hydroxylase-like protein and related to *trans*-zeatin biosynthesis was up-regulated only after benzyl adenine treatment. The DEG encoding gibberellin 20 oxidase 2-like was down-regulated after a benzyl adenine supply while it was up-regulated after a *meta*-topolin supply. Changes in the cytokinin–auxin balance and gibberellin biosynthesis in in vitro shoots may contribute to the morphological differences previously observed for the two cytokinins.

## 1. Introduction

Apple (*Malus x domestica* Borkh.), family Rosaceae, is indisputably the most popular and economically significant fruit crop worldwide, which is widely cultivated in the temperate climate regions of the world [1,2]. It represents a good source of dietary fiber, minerals, vitamin C, folic acid, flavonoids, polyphenols, antioxidants, organic acids, and sugars, making apple consumption of nutritional and medicinal value with beneficial effects on human health [3,4]. It prevents cardiovascular diseases, reduces the risk of diabetes, and also provides anti-asthmatic and anti-allergic effects [5,6]. Significant progress has been made in the genomic study of apples, with high-quality genome accessions available for numerous cultivars and their wild relatives [7].

Cytokinins (CKs) are plant hormones that play a key role in regulating the cell cycle and various developmental processes. Among exogenously aromatic CKs, 6-Benzyl adenine [6-benzylaminopurine] (BA) is the most widely used in commercial micropropagation procedures that have been applied to stimulate de novo shoot regeneration due to their high efficacy in promoting cell division and proliferation as well as to their affordability [8,9]. BA has been the preferred CK for apple shoot multiplication [10,11]. However, since it often has a negative impact on the growth, shoot quality, subsequent rooting and acclimatization of some species, and may also induce other physiological disorders, exploring other alternatives has become necessary [11,12].

*Meta*-topolin [N6-(3-hydroxybenzylamino) purine] (TOP), a hydroxylated BA analog, is a naturally occurring aromatic CK. TOP has been proven to be one of the most effective aromatic CK applied in tissue cultures of various plant species and can be applied successfully in apple micropropagation [11]. It was the most effective CK for in vitro axillary shoot development when compared with BA, benzyladenine-9-riboside (BAR), kinetin (Kin), and thidiazuron (TDZ). TOP could be applied to induce a sufficient multiplication rate and resulted in high-quality in vitro shoots of Húsvéti rozmaring apple scion [3]. It was also superior to other CKs for the shoot induction of other plants [13]. Among the CKs, Kin and BA are usually applied; however, a high potential of TOP has been demonstrated for an improved multiplication rate and in vitro plant quality [14]. In addition, TOP causes better rooting and plant acclimatization. Moreover, TOP is considered not to promote hyperhydricity and could therefore be a substitute for BA, which can induce genetic changes, as well as physiological disorders, including shoot-tip necrosis and hyperhydricity. In addition, TOP was found to be superior in inducing high rates of shoot multiplication as compared to BA [15,16,17,18,19,20,21,22,23]. Furthermore, TOP offers advantages such as minimizing negative side effects that can impair subsequent rooting [24,25,26]. It has been shown to improve shoot regeneration and rooting, and acclimatization efficiency [27,28,29], achieving or ensuring genetic homogeneity [30].

The quality, morphology, and the physiological status of microshoots are of great importance in their subsequent development (adventitious or axillary shoot regeneration, rooting) and acclimatization [3,11]. TOP increased the survival percentage and decreased the hyperhydricity of plantlets regenerated from meristems cultures of the ‘Golden Delicious’ apple [31], enhanced the quality of *Prunus domestica* L. and *Prunus insititia* × *domestica* in vitro shoots [32], induced optimum multiplication rate and achieved high-quality shoots of in vitro grown pear rootstock OHF-333, improved leaf gas exchange, and decreased phenol content [33].

The metabolism of BA and TOP and their effects on the endogenous CKs pool were investigated by evaluating the quantitative and qualitative CK metabolite analyses. The in vitro shoots developed on the TOP-containing medium contained more active CK forms than in the BA-medium, which may explain the superiority of TOP in terms of the proliferation rate [34] and in avoiding hyperhydricity [17]. CK analysis revealed that BA supplementation led to the accumulation of inactivated forms of BA [9], and shoots induced on a medium that contained synthetic cytokinin, such as BA, have accumulated toxic BA metabolites [25]. Consequently, these metabolites influenced shoot development, rooting, and in vitro acclimation of micropropagated plants [25]. However, supplementing the culture medium with natural cytokinins, such as TOP, results in reversibly bound metabolites that play an important role in delaying or reprogramming senescence, enhancing the synthesis of photosynthetic pigments, modulating the antioxidant enzyme activity of cells, and thus improving shoot and root development and subsequent acclimation [35,36].

An earlier study assessed the after-effects of cytokinins BA and TOP added to the shoot multiplication medium on subsequent rooting of an Húsvéti rozmaring apple scion. BA increased the number of roots markedly, while TOP resulted in significantly longer roots [37]. BA had serious side effects both during shoot development and, as harmful after-effects, during either the next propagation cycle (axillary or adventitious), or subsequent rooting and acclimatization processes. These undesirable effects depended on the type and concentration of CKs applied to the shoot proliferation medium and on the plant species/genotypes or explant types [11,38,39]. BA had a negative effect on rooting ability, potentially reducing or inhibiting the formation of roots in many plant species [40,41], inhibiting adventitious root (AR) formation in GL-3 apple microshoots [42].

TOP-treated banana plantlets have demonstrated better acclimatization compared to BA-treated or control plantlets [43]. Moreover, in vitro shoots of apple cv. Húsvéti rozmaring cultured on the TOP-containing medium were more vigorous with larger, dark green leaves and increased shoot length compared to those regenerated on BA [37].

TOP-treated plantlets remained green as reflected by the higher total chlorophyll/carotenoid ratio [44]. They had the best acclimatization ability, which could be due to the high shoot quality at the multiplication stage. Moreover, TOP enhanced chloroplast differentiation, reduced chlorophyll degradation, modified antioxidant enzyme activities, and, as a result, improved rooting and increased the acclimatization capacity [13,45]. TOP application resulted in a well-developed photosynthetic apparatus that enhanced the survival of in vitro plantlets during the acclimatization stage and offered a better acclimatization capability of TOP-regenerants [44]. TOP positively affected the function of the photosynthetic apparatus and increased the pigment content (chlorophyll a/b ratio) of the in vitro leaves of the Royal Gala apple scion, which can help increase the survival rate of plants during acclimatization [46]. It promoted photo-pigments and increased the survival rate of the regenerants [47].

Although the beneficial effects of TOP compared to BA have been well documented in apple shoot multiplication medium, as described in detail above, their effects on gene transcription are still largely unexplored. The aim of this study included the investigation of the transcriptomic response of in vitro shoots of apple cv. Húsvéti rozmaring to the TOP and BA content of the shoot multiplication medium, respectively.

## 2. Results

### 2.1. Evaluation of Global Changes in the RNA Expression Profile

An evaluation of the expression intensity of 51,804 genes, from which 39,340 are protein coding, was performed, including their promoters and coding regions, based on three comparisons according to the CK content of the shoot multiplication medium, such as BA vs. NCK (cytokinin-free, i.e., no cytokinin was added), TOP vs. NCK, and TOP vs. BA. Heat maps were created based on the expression intensity of the genes (Table S1; Figure S1). Volcano plots generated based on CK supply (control, BA, TOP) show up- and down-regulated genes for all comparisons (BA vs. NCK, TOP vs. NCK, and TOP vs. BA) (Figure S2).

**Figure 1:**
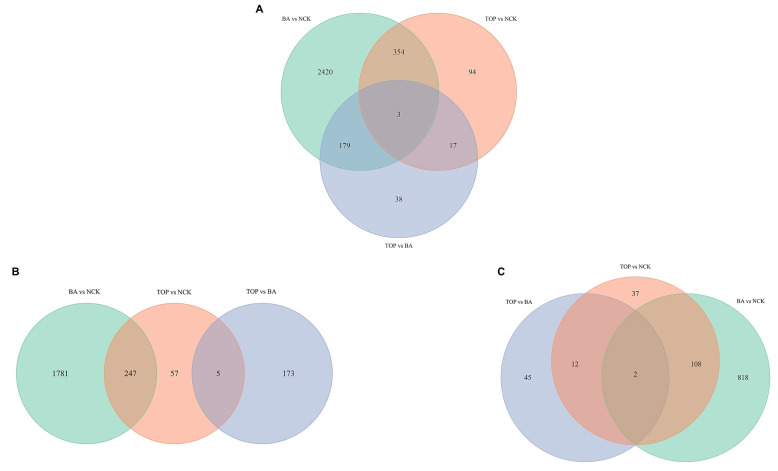
Distribution of significantly differentially expressed genes based on comparisons of BA vs. NCK, TOP vs. NCK, and TOP vs. BA after four weeks of cultivation of in vitro apple (cv. Húsvéti rozmaring) shoots on shoot multiplication media with various cytokinin supplies. (**A**): all DEGs, (**B**): up-regulated DEGs, (**C**): down-regulated DEGs between BA, TOP, and NCK samples (BA: benzyl adenine; TOP: *meta*-topolin; NCK: cytokinin-free).

In comparisons of BA vs. NCK, TOP vs. NCK, and TOP vs. BA, a total of 2956, 468, and 237 genes were significantly differentially expressed, respectively. Considering all DEGs, 354 of them were identical in comparisons of BA vs. NCK and TOP vs. NCK, 17 of them in comparisons of TOP vs. NCK and TOP vs. BA, and 179 of them in comparisons of BA vs. NCK and TOP vs. BA. Furthermore, three DEGs were the same in all three comparisons (Figure 1; Table 1).

When the effects of the BA-containing medium were compared to CK-free medium, about 6.5-fold more up-regulated and 5.8-fold more down-regulated DEGs were detected than when the effects of the TOP-containing medium were compared to the CK-free medium (Table 1). A total of 6.5-fold and 5.8-fold more DEGs were up-regulated than down-regulated due to BA and TOP, respectively, when comparing both to the CK-free medium. When TOP was compared to BA, 3-fold more up-regulated DEGs were recorded than down-regulated ones (Table 1). Of the up-regulated DEGs, 247 were identical in the comparisons of BA vs. NCK and TOP vs. NCK, and 5 in comparisons of TOP vs. NCK and TOP vs. BA. No DEGs could be detected as identical in all three comparisons. Among the down-regulated DEGs, 110 were the same between BA vs. NCK and TOP vs. NCK, 14 between TOP vs. NCK and TOP vs. BA, and 2 between BA vs. NCK and TOP vs. BA. Only two DEGs were identified as identical in all three comparisons (Table S1).

### 2.2. Changes in Biological Processes, Cellular Components, and Molecular Function in Response to Various Cytokinin Supplies

The most important processes related to biological processes, molecular function, and cellular components were assessed based on comparing significant up- and down-regulation in various comparisons. Significantly enriched Gene Ontology terms were identified among the DEGs. A cut-off value of LFC ≥ 1 of DEGs was generally applied in each comparison to assess the most weighted (important) processes and functions (Table S2; Figure S3).

In the comparison between BA and NCK, a total of 64 biological processes, while between TOP and NCK 32 biological processes, were affected. The vast majority of significantly up- and down-regulated DEGs in the comparison of BA vs. NCK belonged to the following 20 biological processes: aromatic amino acid family catabolic process, carbohydrate metabolic process, erythrose 4-phosphate/phosphoenolpyruvate family amino acid catabolic process, establishment of localization, localization, folic acid-containing compound metabolic process, L-phenylalanine catabolic process, L-phenylalanine metabolic process, transport, biological regulation and regulation of various processes like biological process, biosynthetic process, cellular biosynthetic process, cellular metabolic process, cellular process, gene expression, macromolecule biosynthetic process, macromolecule metabolic process, metabolic process, and RNA metabolic process.

The involved molecular functions can be linked to a total of 20 categories (hydrolase activity, transporter activity, transmembrane transporter activity, phosphoric ester hydrolase activity, carbohydrate/proton symporter activity, carbohydrate/monoatomic cation symporter activity, secondary active transmembrane transporter activity, symporter activity, solute/monoatomic cation symporter activity, solute/proton symporter activity, oxidoreductase activity acting on NAD(P)H, ATP hydrolysis activity, ATP-dependent activity, hydrolase activity acting on ester bonds, isopentenyl-diphosphate delta-isomerase activity, hydrolase activity hydrolyzing o-glycosyl compounds, sulfuric ester hydrolase activity, oxidoreductase activity acting on NAD(P)H quinone or a similar compound as acceptor, phosphatase activity, active transmembrane transporter activity, and active monoatomic ion transmembrane transporter activity), and DEGs were limited to only one location, i.e., the nucleus (Table S2; Figure S3).

In the comparison between TOP and NCK, the majority of significantly up- and down-regulated DEGs belonged to 32 groups, the 20 most important groups being cell wall organization, cell wall organization or biogenesis, DNA-templated transcription, external encapsulating structure organization, plant-type cell wall organization, plant-type cell wall organization or biogenesis, regulation of various processes like biological process, biosynthetic process, cellular biosynthetic process, cellular metabolic process, cellular process, DNA-templated transcription, gene expression, macromolecule biosynthetic process, macromolecule metabolic process, metabolic process, nucleobase-containing compound metabolic process, primary metabolic process, RNA biosynthetic process, and RNA metabolic process. Only one molecular function was annotated, the DNA-binding transcription factor activity. Cellular components were annotated in two locations, i.e., the cell wall and external encapsulating structure (Table S2; Figure S3).

### 2.3. Transcription Factor Behavior in Response to Different Cytokinin Supply

The identification of DEGs related to transcription factors was determined using the NCBI database (Table S1) and the literature data of [48,49,50].

**Figure 2:**
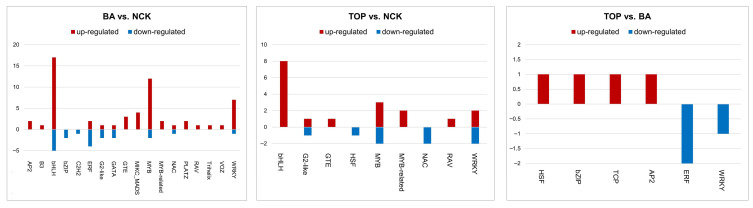
Transcription factors (TFs) affected by cytokinin supply of the shoot multiplication media four weeks after culturing on them. TFs are grouped based on TF families in each comparison of treatments (BA; TOP) vs. NCK (control) and both cytokinin treatments to each other (BA: benzyl adenine; TOP: *meta*-topolin). The bar charts represent the number of TFs belonging to each TF family.

Comparing BA-treated plants with the control group (NCK), 17 TFs were up-regulated and 5 were down-regulated in the bHLH (basic helix-loop-helix) TF family. When comparing TOP vs. the control group, eight bHLH TFs were up-regulated. In the case of in vitro shoots cultivated on BA-containing media for four weeks, two ERF (ethylene-responsive factor) TFs were up-regulated and four were down-regulated compared to the control group. In contrast, when comparing the two cytokinin treatments, only one TF belonging to the bHLH family was up-regulated and two TFs belonging to the ERF TF family were down-regulated (Figure 2; Table S1).

Concerning the G2-like (Golden2-like) family, we found that in the BA vs. control comparison, MYBR7 was up-regulated and two TFs were down-regulated. MYBR7 was up-regulated also when comparing TOP with the control, and in this case, one TF was down-regulated.

In the case of BA vs. control, only one TF from the GATA TF family was up-regulated and two were down-regulated. In the same comparison, three members of the GTE (Global Transcription Factor Group E) TF family were up-regulated, while only one was up-regulated in the case of TOP. As a result of BA treatment, four TFs of the MIKC_MADS (MIKC-type MADS) TF family were up-regulated, while this could not be detected in the comparisons of the other treatments (Figure 2; Table S1).

MYB-related transcription factors comprise a large subfamily of the MYB family. When comparing in vitro shoots grown on the BA-containing medium with control plants, we found that 14 TFs were up-regulated, and 2 were down-regulated. When plants were treated with TOP, five were up-regulated, and two were down-regulated, while when comparing the two treatments, no TFs from this family were observed.

From the WRKY family, seven TFs were up-regulated and one was down-regulated when BA was added, in the case of TOP, only two were up-regulated and two were down-regulated, while when comparing the two groups, only one TF was down-regulated in this family.

When examining TOP vs. BA, it was also observed that one TF was up-regulated in the HSF (Heat Shock), TCP, and AP2 (APETALA2) TF families, respectively (Figure 2; Table S1).

### 2.4. Up- and Down-Regulated DEGs Related to Auxin Signaling and Transport

In plants, one of the primary coordinating signals is the hormone auxin (indole-3-acetic acid; IAA), which regulates plant growth and development via essential cellular processes, such as cell division, expansion, and differentiation [51].

**Figure 3:**
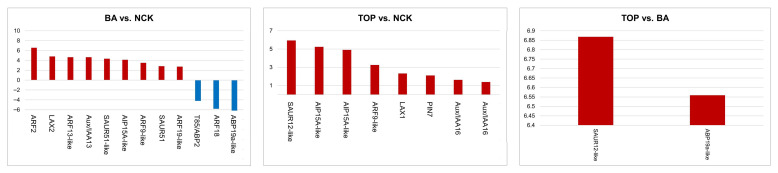
Differentially expressed genes (DEGs) related to auxin transport and signaling in response to cytokinin supply in the shoot multiplication medium four weeks after cultivation, in each comparison of treatments (BA; TOP) vs. NCK (control) and both cytokinin treatments to each other (BA: benzyl adenine; TOP: *meta*-topolin). The bar charts show the logarithmic fold change (LFC) values for auxin-related genes.

In total, 12, 8, and 2 auxin-related genes changed their transcriptional level in the BA vs. NCK, TOP vs. NCK, and TOP vs. BA comparison, respectively (Figure 3; Table S1).

In the case of BA vs. NCK, 12 auxin-associated DEGs were identified, including 9 up-regulated and 3 down-regulated genes. We observed that four auxin response factors (ARF2, ARF13-like, ARF9-like, and ARF19-like) DEGs were up-regulated, and one (ARF18) was down-regulated. Two auxin-binding proteins (T85/ABP2 and APB19a-like) also decreased in their expression. Moreover, the expression of two protein small auxin up-regulated RNAs (SAUR51 and SAUR51-like), the auxin-induced protein 15A-like (AIP15A-like) [52], and the auxin-responsive protein IAA13 were up-regulated. Lastly, the auxin transporter-like protein 1 (LAX2) increased its mRNA level as well (Figure 3; Table S1).

In the comparison between TOP and NCK, all the auxin-related genes found are up-regulated. The protein small auxin up-regulated RNA 12-like (SAUR12-like) DEG was observed. Two auxin-induced proteins 15A-like (AIP15A-like) DEGs were found to change expression. Three genes such as the auxin response factor (ARF9-like) and two auxin transport-linked genes, the auxin transporter-like protein 2 (LAX1) and the auxin efflux carrier component 3 (PIN7), increased their mRNA level. In addition, two auxin-responsive protein IAA16s were detected to change their expression (Figure 3; Table S1).

When comparing differences between TOP-treated and BA-treated plants, two auxin-related DEGs are up-regulated. One is the protein small auxin up-regulated RNA 12-like (SAUR12-like), and the other is the auxin-binding protein ABP19a-like DEG (Figure 3; Table S1).

### 2.5. KEGG Mapping of up- and Down-Regulated DEGs Related to Metabolic and Cellular Processes

After the shoots were cultured on BA-containing medium for four weeks, more than 5-fold more DEGs were detected during KEGG mapping than after shoots were cultured on TOP-containing medium (i.e., 27 DEGs in BA vs. NCK, and 5 DEGs in TOP vs. NCK) when the expression intensity of genes was compared to shoots grown on the CK-free, NCK medium. DEGs related to valine, leucine, and isoleucine degradation in shoots grown on the BA-containing medium were down-regulated while DEGs related to the metabolic pathways and biosynthesis of secondary metabolites were both up- and down-regulated. Efferocytosis was down-regulated in the TOP vs. BA comparison (Table 2; Table S3; Figure S4).

Valine, leucine, and isoleucine degradation was attenuated via the down-regulation of two DEGs (aldehyde dehydrogenase family 2 member B4, mitochondrial, ALDH2B4, EC1.2.1.3, and 3-ketoacyl CoA thiolase, peroxisomal, KAT, EC 2.3.1.16) (Table 2; Tables S3 and S4; Figure S4).

The biosynthesis of secondary metabolites was elevated in shoots grown on the BA-containing medium by two DEGs (homogentisate phytyltransferase 1, chloroplastic-like, HPT1, EC 2.5.1.115 and 1,2-diacylglycerol kinase 1-like ATP-dependent, DGK1, EC 2.7.1.107). On the other hand, the biosynthesis of secondary metabolites was down-regulated by eight DEGs: succinate dehydrogenase (ubiquinone) flavoprotein subunit (SDHA, mitochondrial, EC 1.3.5.1), mannose 6-phosphate isomerase 1-like (PMI, EC 5.3.1.8), chorismate mutase 1 (CM1, EC 5.4.99.5), proline dehydrogenase 2 (PRODH2, EC 1.5.5.2), gibberellin 20 oxidase 2-like (GA20ox2-like, EC 1.14.11.-), 2,3-bisphospoglycerate-independent phosphoglycerate mutase (iPGAM, EC 5.4.2.12), aldehyde dehydrogenase family 2 member B4, mitochondrial (ALDH2B4, EC 1.2.1.3), and 3-ketoacyl CoA thiolase, peroxisomal (KAT, EC 2.3.1.16) (Table 2; Tables S3 and S4; Figure S4).

The BA content of the medium led to altered gene expression intensity in DEGs related to metabolic pathways. Three DEGs were up-regulated (homogentisate phytyltransferase 1, chloroplastic-like (HPT1, EC 2.5.1.115), 1,2-diacylglycerol kinase 1-like ATP-dependent (DGK1, EC 2.7.1.10 and V-type proton ATPase subunit E1 (ATP6V1E1, EC 3.6.3.14)), while twelve DEGs were down-regulated (succinate dehydrogenase (ubiquinone) flavoprotein subunit (SDHA, mitochondrial, EC 1.3.5.1), mannose 6-phosphate isomerase 1-like (PMI, EC 5.3.1.8), chorismate mutase 1 (CM1, EC 5.4.99.5), proline dehydrogenase 2 (PRODH2, EC 1.5.5.2), gibberellin 20 oxidase 2-like (GA20ox2-like, EC 1.14.11.-), 2,3-bisphospoglycerate-independent phosphoglycerate mutase (iPGAM, EC 5.4.2.12), aldehyde dehydrogenase family 2 member B4, mitochondrial (ALDH2B4, EC 1.2.1.3), 3-ketoacyl CoA thiolase, peroxisomal (KAT, EC 2.3.1.16), quinolinate phosphoribosyltransferase (QRTP, EC 2.4.2.19), photosystem I reaction center subunit XI, chloroplastic-like (PsaL, EC 1.97.1.12), glutathione reductase, cytosolic (GR, EC 1.8.1.7), glutathione S-transferase f12-like/(GST, EC 2.5.1.18)) (Table 2; Tables S3 and S4; Figure S4).

In shoots grown on the TOP-containing medium both up- and down-regulated DEGs were detected, and all of them were related to metabolic pathways. Only one DEG was up-regulated, which encodes magnesium protoporphyrin IX methyltransferase (ChlM, EC 2.1.1.11), while DEGs related to NAD(P)H dehydrogenase (quinone) FQR1-LIKE (EC 1.6.5.2), quinolinate phosphoribosyltransferase (QPRTASE, EC 2.4.2.19), glutathione reductase, cytosolic (GR, EC 1.8.1.7), and 2,3-bisphosphoglycerate-independent phosphoglycerate mutase (iPGAM, EC 5.4.2.12) were down-regulated (Table 2; Tables S3 and S4; Figure S4).

In the TOP vs. BA comparison, three DEGs were up-regulated. Two of them were related to metabolic pathways of linoleic acid metabolism (probable linoleate 9s-lipoxygenase 5, LOX5, EC 1.13.11.58), and diterpenoid biosynthesis (gibberellin 20 oxidase 2-like GA20ox2-like, EC 1.14.11.-). The third up-regulated DEG encoding ras-related protein RABF1, which is a small GTPase in plants, was related to a pathway of cellular transport and catabolism (Table 2; Tables S3 and S4; Figure S4).

### 2.6. Validation with RT-qPCR

It is assumed that the expression of the reference genes remain relatively constant under different experimental conditions. The most suitable housekeeping gene was considered to be GAPDH, which would allow an accurate comparison of gene expression levels.

**Figure 4:**
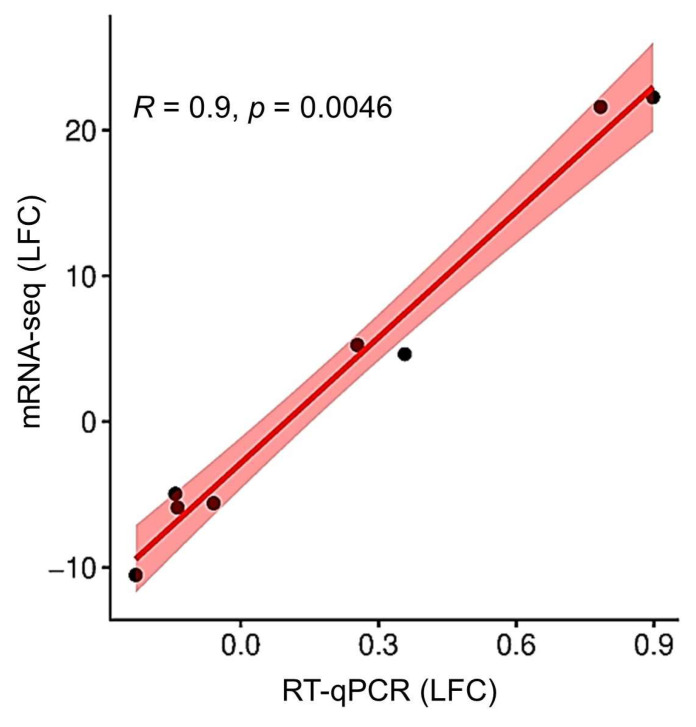
Validation of mRNA-seq data by RT-qPCR using Spearman correlation coefficient. The correlation was calculated and illustrated via SRplot. The dotted line represents the ideal scenario where all data points fall exactly on the line, indicating a perfect linear relationship.

The results obtained from both mRNA-seq and RT-qPCR showed the same direction—either up- or down-regulation of differential expression and differential expression logarithmic fold change (LFC) values of the target genes. The Spearman correlation coefficient was 0.9 (Table S5, Figure 4). This high correlation coefficient shows a strong positive correlation between the mRNA-seq LFC and RT-qPCR LFC (Table S5; Figure 4).

## 3. Discussion

Using TOP as the cytokinin source in the shoot multiplication medium instead of BA has been proven to have several beneficial effects, such as increasing the multiplication rate, functional and ultrastructural improvement of in vitro leaves, supporting subsequent in vitro rooting, and acclimatization efficiency in various plant species [25,43,53,54,55,56]. Its use in the apple shoot multiplication medium was reported in various scion and rootstock cultivars. TOP increased the ratio of chlorophyll-a/chlorophyll-b compared to BA in apple scion cvs. Royal Gala and Freedom [46,57]. The best shoot multiplication rate was detected after the application of TOP in apple rootstock of JTE-H and scion cv. Jonagold [58,59]. In cv. Húsvéti rozmaring, the most effective multiplication rate and the highest shoot quality was achieved when TOP was the cytokinin-source in the shoot multiplication medium [3]. In the present study, the transcriptomic replies of apple shoots cultured in vitro were revealed either at the BA or TOP supply of shoot multiplication media.

### 3.1. Global Changes in the RNA Expression Profile

RNA-seq analysis revealed that about 6-fold more significantly up-, or down-regulated DEGs could be detected in shoots grown on the BA-containing medium than in those grown on the TOP-containing medium when comparing both to the control shoots grown on the cytokinin-free medium (Figure 1; Table 1; Table S1). An evaluation of the most significant changes in biological processes in response to different cytokinin supplies, i.e., BA or TOP supply, revealed that approximately half of the most important biological processes affected were identical. All of them were related to regulatory processes, such as the regulation of biological, biosynthetic, cellular biosynthetic, cellular, macromolecule biosynthetic, macromolecule metabolic, metabolic, and RNA metabolic processes as well as gene expression (Table S2; Figure S3). The two plant growth regulators tested had different effects on cellular components: BA affected the cell nucleus, and TOP affected the external components, i.e., the cell wall and the outer capsule structure. Regarding molecular functions, 20 functions were enriched in response to BA, but only 1, the activity of the DNA-binding transcription factor, was enriched in response to TOP, the latter of which was not observed between the enriched molecular functions of shoots grown on BA.

### 3.2. The Transcription Factors Affected Most in Response to Different Cytokinin Supply

The most affected TFs included WRKYs, bHLH, and MYB after both CK supplies, while MIKC-type MADS-box TFs, ERF, and AP2 TFs were affected in response to BA supply. WRKYs may act as master regulators balancing plant growth with responses to biotic and abiotic stress. These genes are responsible for the modulation of transcription related to plant defense responses to environmental stressors such as a rapid increase in reactive oxygen species, Ca^2+^ influx, mitogen-activated protein kinase activation, phytohormone production, and epigenetic modification [60]. WRKY TFs were detected to be in all comparisons in response to exogenous BA or TOP supply (Figure 2). Plant bHLH transcription factors play a role in growth and development processes [61]. The bHLH proteins also affect metabolism, biosynthesis, and signal transduction, including anthocyanin synthesis, light signaling, and brassinosteroid signaling [62,63,64]. We observed up- and down-regulated bHLH TFs when adding BA, and up-regulated ones when adding TOP (Figure 2). MYB TFs are central regulators in several different plant-specific processes, including phenylpropanoid metabolism, cell cycle, root hair and trichome formation, phytohormones responses, reproductive growth, and responses to abiotic or biotic stress [65]. We observed differently expressed TFs from the MYB family and its MYB-related subclass under both cytokinin treatments (Figure 2). MIKC-type MADS-box genes take part in practically every aspect of plant development [66]. There have also been reports of their involvement in different stress responses [67,68,69]. When the medium contained BA, the expression intensity of MIKC-type MADS-box TFs was increased (Figure 2). The expression intensities of TFs from the ERF and AP2 subfamily were also altered when the medium contained BA (Figure 2). The ERF/AP2 TFs are involved in abiotic stress responses mediated by gibberellins, auxins, brassinosteroids, and cytokinins. They are responsible for the regulation of the abiotic stress responses of plants and can also partake in the regulation of plant growth and development [70].

### 3.3. DEGs Related to Auxin Signaling and Transport

**Figure 5:**
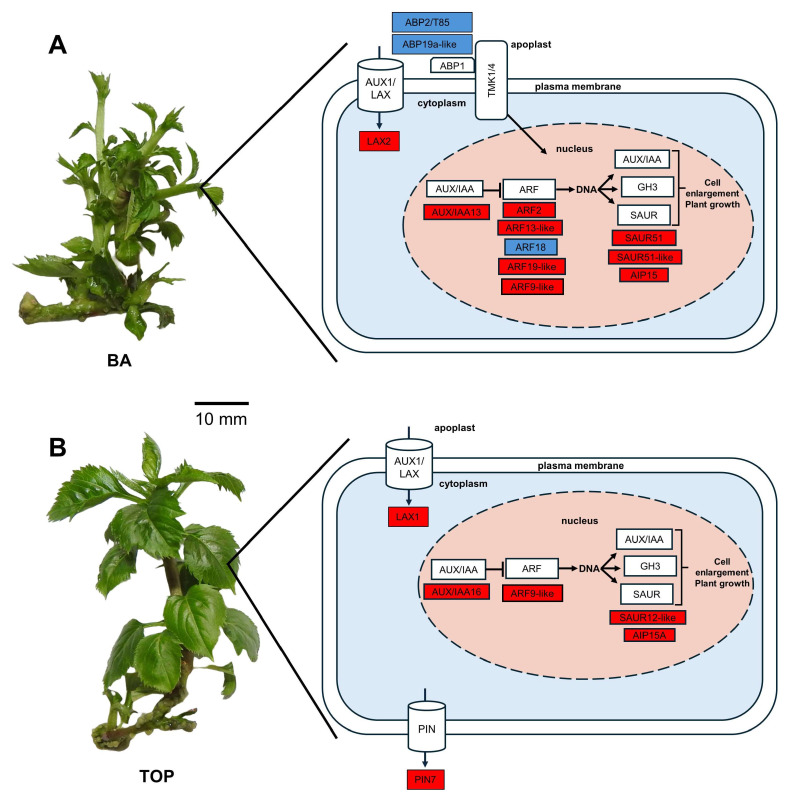
Putative roadmap of up-regulated (red boxes) and down-regulated (blue boxes) DEGs related to auxin transport and signaling in response to BA (**A**) and TOP (**B**) treatments. Extracellular auxin is perceived by ABPs and transported by AUX1/LAX1 and PIN transmembrane proteins. In the nucleus, AUX/IAAs repress transcriptional activation of ARFs. When the Aux/IAA repression is terminated, they can recover ARF activity and activate transcription of auxin-responsive genes.

The CK content of the culture medium significantly influenced the expression of genes related to auxin signaling and auxin transport, depending on the type of CK (Figure 5). As per the observations of Jones et al. [71] an increase in auxin synthesis was induced by treatment with various cytokinins in young leaves, the shoot apex, and the root system. Concerning cytokinin, it was also found by Laplaze et al. [72] that it regulates auxin signaling and, in particular, auxin transport. Růžička et al. [73] presented further evidence in their study that cytokinin modulates auxin transport by regulating PIN transporters, by showing that cytokinin decreases the expression of PIN1 and PIN3 in the *Arabidopsis* root meristem while increasing that of PIN7. Our results indicated that in the case of TOP-treated plants, PIN7, the auxin efflux carrier component 3 was up-regulated. The auxin transporter families include auxin influx carriers (AUX1/LAX), auxin efflux carriers (PIN), ATP-binding cassette-B (ABCB)/P-glycoprotein (PGP), and auxin transporters from intracellular spaces (PIN-LIKES, PILS) [74,75]. PIN-FORMED (PIN) proteins are a plant-specific family of transmembrane proteins transporting auxin, a plant signal molecule, as their substrate. Of those characterized, the most are located in the plasma membrane [76]. According to our observations, additional auxin transporter-like proteins, LAX1 (BA vs. NCK) and LAX2 (TOP vs. NCK) were overexpressed as well. In *Arabidopsis thaliana*, LAX2, a member of the AUX1/LAX family, regulates vascular patterning in cotyledons. The aerial development of *Arabidopsis* requires LAX1 and LAX2. The latter regulates vascular development, and both are required for leaf phyllotactic patterning [77,78].

To control the expression of auxin response genes, two types of transcription factor families are required: the auxin response factor (ARF) family and the Aux/IAA repressor family [79]. ARF binds to auxin response DNA elements (AuxRE) in promoters of primary or early auxin-responsive genes, such as Aux/IAA, Gretchen Hagen3 (GH3), and small auxin-up RNA (SAUR) family members. They thus act as a transcription factor regulating the expression of auxin response genes [80]. ARFs are key components in the auxin signaling pathway that are known to regulate cellular growth and development processes under normal cellular conditions [81]. When we compared BA vs. NCK, we observed four up-regulated (ARF2, ARF13-like, ARF9-like, ARF19-like) and one down-regulated (ARF18) auxin response factor DEGs. In the case of TOP vs. NCK, one ARF9-like DEG was up-regulated. The transcription factor ARF2 acts as a positive activator of flowering senescence and abscission, while it represses cell growth in the presence or absence of light, and differential hypocotyl growth [82]. ARF9 (SlARF9) negatively controls cell division during the early development of fruit in *Solanum lycopersicum* [83]. ARF13, which contains a repression domain and is localized to the nucleus in *M. domestica*, acts as a negative regulator of the anthocyanin metabolic pathway through Aux/IAA–ARF signaling [84]. Repression of the auxin signaling repressor ARF18 intensifies auxin signaling and promotes the elongation of the hypocotyl in *A. thaliana* [85]. ARF7 and ARF19 contribute significantly to the IAA7-mediated growth and development of *Arabidopsis*, including sensitivity to auxin and root gravitropism, the formation and elongation of root hairs, and the formation of lateral roots [86].

If the levels of auxin are low, interacting with ARFs, Aux/IAA (auxin-responsive) proteins inhibit the activation of ARF target genes [87]. In the comparison between BA and NCK, an auxin response protein, Aux/IAA13 was found to have increased its expression. In the case of TOP vs. NCK, two auxin response proteins, Aux/IAA16, were up-regulated. In *Acer rubrum*, the main inhibitors of downstream gene transcription of ARFs are Aux/IAA13 and Aux/IAA16, which interact with ARFs in the nucleus. This also indicates their involvement in the regulatory function of key regulatory pathways involved in the signal transduction of plant hormones in relation to root growth and development [88].

Early auxin-responsive gene families include, e.g., small auxin up-regulated RNAs (SAURs) [89]. The genes with the most rapid response to auxin that are related to the auxin signaling pathway can be found in the SAUR gene family [90]. SAURs may be regulated at the transcriptional, post-transcriptional, or protein level [91,92]. Up-regulated SAURs were found in all three comparison groups. The mRNA level of SAUR51 and SAUR51-like genes was increased when applying BA treatment. The expression of SAUR12-like genes was also increased in the case of TOP treatment. When comparing differences between TOP-treated and BA-treated plants, the SAUR12-like DEG was up-regulated. In *A. thaliana*, SAUR51 is induced specifically by auxin, with expression in the root tips and expanding leaves, implying the general importance of SAUR genes for cell elongation [93].

A class of low-abundance proteins in plants, auxin-binding proteins (ABPs) bind active auxins with high specificity and affinity. ABP could possibly initiate the auxin signal pathways, resulting in various cellular responses via ABP-auxin binding, in accordance with a plant hormone receptor function [94]. Our observations revealed two down-regulated auxin-binding proteins (T85/ABP2 and ABP19a-like) in the BA-treated group, while on the contrary, the ABP19a-like was up-regulated in the BA vs. TOP comparison.

The different effects of BA and TOP supply of the culture medium on the expression intensity of genes involved in auxin signaling and auxin transport processes (Figure 5) may influence the auxin/cytokinin ratio of in vitro shoots, and thus cell elongation, growth, and division in them. Furthermore, in the comparison of BA vs. NCK, but not in TOP vs. NCK comparison, a DEG encoding cytokinin hydroxylase-like protein was up-regulated (LFC: 5.96) (Table S1). Cytokinin hydroxylase can catalyze the biosynthesis of trans-Zeatin [95], which could further influence the cytokinin–auxin balance of the in vitro shoots.

### 3.4. DEGs Related to Metabolic and Cellular Processes Influencing Redox and Hormonal Balances

Considering the results of KEGG mapping, DEGs related to auxin synthesis or signal transduction could be mapped. Plant ALDHs are involved in the metabolism of aldehydes, ketones, and acids. Furthermore, they may be linked to the synthesis of plant hormones like auxin. ALDH2B4 catalyzes the oxidation of toxic aromatic/aliphatic aldehydes to non-toxic carboxylic acids, thus it has a potential role in the pyruvate dehydrogenase (PDH) bypass pathway, which can function as an alternative to the normal PDH complex. ALDH2B4, that was down-regulated in the case of BA vs. NCK, is involved in the biosynthesis of secondary metabolites and metabolic pathways, different metabolic pathways, and also valine, leucine, and isoleucine degradation. While ALDH2B4 is constantly expressed in plants, their down-regulation may impair their ability to respond to certain environmental stresses (particularly oxidative stress), as it may cause the accumulation of aldehydes. Thus, they could indirectly affect growth and development in a way that the altered regulation of ALDH2B4 gene expression might influence auxin levels [96,97]. KAT, which was also down-regulated in the case of BA vs. NCK, is involved in degradation pathways including fatty acid beta-oxidation via performing the reverse Claisen condensation reaction, thus providing energy and substrates (including precursors for auxin metabolites) for proper growth and development. The phenotypes of KAT knockout mutants, with altered growth and development (the processes of which are heavily influenced by auxin), are similar to those seen in some auxin-related mutants [98,99]. However, the direct links or mechanisms have not been demonstrated in research.

HPT1 is involved in tocopherol metabolism and biosynthesis via catalyzing the condensation of homogentisate and phytyl diphosphate to form dimethylphytylhydroquinone. The gene expression intensity of HPT1, an enzyme that helps plants adapt to various stress conditions, was up-regulated in the case of BA vs. NCK [100]. Meanwhile, DGK1, which was up-regulated in the BA vs. NCK comparison, plays a role in glycerophospholipid metabolism (lipid signaling) by catalyzing diacylglycerol (DAG) phosphorylation to generate phosphatidic acid (PA). In this way, PA production coupled with the function of DGK1 can modulate the activation of many phytohormones and other cellular processes (stomatal closure, cell membrane permeability regulation, etc.) by altering its enzyme activity [101].

A flavoprotein subunit, namely succinate dehydrogenase (ubiquinone) flavoprotein subunit (SDHA, mitochondrial, EC 1.3.5.1), also known as Complex II acts as the initial electron acceptor during the oxidation of succinate to fumarate, transferring electrons to ubiquinone. In the case of BA vs. NCK comparison, the gene expression intensity of SDHA was down-regulated. Reduced mRNA levels of SDHA can cause an increased stomatal aperture and number, leading to greater water loss but also higher photosynthetic rates and enhanced plant growth. Furthermore, its down-regulation may disrupt normal cellular energy metabolism, which in turn causes a metabolic and ROS-mediated signaling cascade that results in higher free auxin concentrations and enhanced sensitivity to the hormone. The altered mitochondrial functions and changes in ROS levels also affect the expression of auxin-related genes including Auxin Response Factors (ARFs) and GH3 genes, which conjugate free auxin [102].

The reduced amount of mannose 6-phosphate isomerase 1-like (PMI, EC 5.3.1.8) in the case of BA vs. NCK is toxic, since it impairs glycolysis and therefore ATP production by the accumulation of mannose-6-phosphase [103]. In the shikimate pathway, chorismate mutase 1 (CM1, EC 5.4.99.5) is a pivotal branch point enzyme, channeling chorismate into the synthesis of phenylalanine and tyrosine. Furthermore, it also contributes to the biosynthesis of lignin, salicylic acid, and anthocyanins. The down-regulation of genes encoding CM1 (in the BA vs. NCK context) can lead to significant metabolic changes including altered lignin content and shifts in xylem structure. In addition, it can cause increased auxin levels as chorismate would be shunted away from the phenylalanine/tyrosine pathway (which CM1 initiates) and instead be more available for the tryptophan-dependent pathway (higher levels of Trp results in increased production of free IAA) [104].

Plants typically reduce proline dehydrogenase 2 (PRODH2, EC 1.5.5.2) activity under stress conditions leading to the accumulation of proline and reduction in growth by hindering ATP production because proline oxidation feeds into the mitochondrial electron transport chain. In the BA vs. NCK comparison, PRODH2 was down-regulated. The outcome of the down-regulation of the gibberellin 20 oxidase 2-like (GA20ox2-like, EC 1.14.11.-) gene is active GA deactivation, reduction in their accumulation, and promoting developmental processes like phase transition from vegetative to reproductive growth, leading to semi-dwarfism in plants. The gene expression intensity of GA20ox2-like was down-regulated in BA which caused a visible height difference compared to the effects of TOP, where this gene was up-regulated [105].

In response to abiotic stresses like drought, cold, or salt treatments, the up-regulation of V-type proton ATPase subunit E1 (ATP6V1E1, EC 3.6.3.14) increases salt and mannitol tolerance, establishing the enzyme’s role in pH homeostasis, vesicular trafficking, cell expansion, and stomatal aperture regulation [106]. The gene expression intensity of ATP6V1E1 was up-regulated in apple shoots on the BA-containing medium compared to the NCK treatment group.

Maintaining redox homeostasis is important during normal growth and development [107]. Different CK supplies in the culture medium differentially affected the expression of genes related to the maintenance and restoration of redox homeostasis in plants. In response to various stressors (e.g., exogenously applied BA), down-regulation of GSTs can occur leading to increased oxidative stress by impairing the plant’s ability to detoxify reactive oxygen species and other electrophiles [108]. Another enzyme in the glutathione system was down-regulated in response to BA in the medium, namely glutathione reductase (GR). It leads to a less reduced glutathione pool, an imbalance in the redox state, and possible damage to cellular components and developmental arrest [109]. In the case of BA, down-regulation of PsaL hinders the electron transfer efficiency through the photosystem, thus impacting the NADP^+^ reduction and overall photosynthetic performance. Furthermore, PsaL plays a role in the stabilization of PSI complexes as its down-regulation reduces its abundance to other subunits [110]. The down-regulation or disruption of QPRTase significantly impairs the de novo synthesis of NAD^+^ and compromises the defense mechanisms against oxidative stress and growth pattern [49]. In both the BA vs. NCK and the TOP vs. NCK comparisons, the expression intensity of QPRTase was down-regulated. ChlM, which was up-regulated in the TOP treatment group, catalyzes the transfer of a methyl group from S-adenosylmethionine to magnesium protoporphyrin IX, a step that forms magnesium protoporphyrin IX monomethyl ester (MgPME) and is necessary for chlorophyll formation. The disruption of the chlorophyll pathway or adverse environmental conditions may cause the up-regulation of ChlM to aid in repair [111]. As FQR1-like has a role in the detoxification and maintenance of redox homeostasis, stress conditions or chemical exposures trigger a shift in the plant’s response, affecting the expression of FQR1. Consequently, its down-regulation in case of TOP vs. NCK may lead to the attenuation of electron transfer from NADH and NADPH to various quinones [112]. As part of the plant defense system, during oxidative stress and lipid peroxidation, LOX5 may play an antagonistic role in ethylene signaling, and is involved in various aspects of plant physiology, growth, and development, as well as senescence, or response to wounding [113]. RABF1 up-regulation is observed under abiotic stress and senescence, where it functions as a molecular switch during vesicular trafficking which involved budding, targeting, and fusion of vesicles within the cell [114]. Both the LOX5 and RABF1 genes were up-regulated in the TOP vs. BA comparisons.

## 4. Materials and Methods

### 4.1. Plant Material, In Vitro Growing Conditions, and Sample Collection

For the experiments, in vitro maintained shoot cultures of apple (*Malus x domestica* Borkh. cv. Húsvéti rozmaring) served as explant source. These cultures were maintained on MS medium (containing MS macro and micronutrients and vitamins [115] supplemented with 0.49 μM indole butyric acid (IBA), 2.22 μM BA, 0.58 μM gibberellic acid (GA_3_), 3% (*w*/*v*) sucrose, and 0.7% (*w*/*v*) agar-agar. The pH of the medium was adjusted to 5.8 before autoclaving at 121 °C and 1.2 bar pressure for 20 min. Each culture vessel contained five shoot explants and 70 mL of medium. Shoot cultures were grown under controlled conditions inside a culture room with a 16/8 photoperiod, at a light intensity of 106 µmol s^−1^ m^−2^ provided by a 1:1 ratio of daylight and warm white fluorescent lamps at a temperature of 23 ± 2 °C. In order to gather an adequate amount of plant material for the experiments, after four weeks of cultivation, shoot explants were transferred onto fresh media. However, to avoid the after-effects of the previous cytokinin supply in the maintaining medium, for the last subculture before the experiments, a cytokinin-free medium was used.

After four weeks of culture on the cytokinin-free medium, individual shoots were excised from the in vitro mother shoots (shoot clusters), then cut into 20 mm long segments. Five explants were transferred into a culture vessel containing the experimental media (70 mL medium/vessel). The preparation and composition of the experimental media was almost identical to that of the medium used for the maintenance described above, except for the CK content. Three different CK-containing media were used during the experiments: a medium without CK (no-cytokinin control = NCK), media containing 4.5 μM TOP, and media containing 4.5 μM BA, respectively. The culture conditions were completely identical to those outlined for in vitro plant maintenance. At the end of the subculture, on the fourth week, apple shoots were collected as samples from three different culture vessels from each CK treatment. Collected samples were immediately chilled in liquid nitrogen and stored at –80 °C until RNA isolation.

### 4.2. Isolation of mRNA and Sequencing

Based on the manufacturer’s protocol, total RNA was purified from three samples per treatment group as three biological replicates each using the Quick-RNA^TM^ Plant Miniprep kit (Zymo Research, Irvine, CA, USA). Isolated RNA samples were assessed via microcapillary electrophoresis using an Implen n50 nanophotometer (Implen, Munich, Germany) and by fragment analysis with an Agilent Bioanalyzer 2100 system using RNA 6000 Nano kit (Agilent, Santa Clara, CA, USA) to ensure adequate quantity and quality.

Following mRNA library preparation protocol (poly A enrichment and cDNA library construction), to sequence the samples using the Illumina Sequencing Platform of the NovaSeq X Plus Series (PE150), the paired-end 150 bp strategy was applied. Under BioProject accession PRJNA1358718, all raw sequences have been deposited into the NCBI Sequence Read Archive (SRA) repository.

### 4.3. Bioinformatic Analysis and Functional Annotation of the Dataset

Raw sequencing data were processed to remove adapters using fastp (v0.26.0) [116] and quality-assessed with FastQC (v0.12.1) [117]. Reads were aligned to the GDT2T_hap1 (GCF_042453785.1) *Malus domestica* reference genome assembly using Hisat2 (v2.1.1) [118]. A quantification of aligned reads was performed with FeatureCounts (v2.1.1) software [119]. Data were normalized and clustered, and differentially expressed genes (DEGs) were identified using DESeq2 (v1.44.0) [120], with a threshold of log2-fold change > 1 and an adjusted *p*-value < 0.05.

Functional enrichment analysis of differentially expressed genes was performed using the ClusterProfiler package (v4.12.6) [121]. To detect significantly enriched Gene Ontology (GO) terms and KEGG pathways, a significance threshold of adjusted *p*-value < 0.05 was applied. Visualizations were generated using the ggplot2 package (v3.5.2) [122] within the R programming environment (v4.4.1) [123].

### 4.4. RT-qPCR Analysis for Validation

Total RNA was isolated and purified, and the quality of the samples was assessed as mentioned before in the subsection ‘Isolation of mRNA and sequencing’.

mRNA samples were diluted to 200 ng/μL. Afterwards, cDNA was synthetized from these samples with 2.5 μM of Random nonamers using the Reverse Transcriptase Core kit (Eurogentec, Searing, Belgium) following the manufacturer’s instructions. The reaction consisted of three steps, the initial, incubation step was 10 min at 25 °C, followed by the reverse transcriptase step of 48 °C for 30 min, then, in the last step, the RT enzyme was inactivated at 95 °C for 5 min. All cDNA samples were stored at −20 °C until further analysis.

Two reference/housekeeping genes were selected based on previous studies, the protein coding actin gene (LOC103445585) and glyceraldehyde-3-phosphate dehydrogenase (GAPDH, LOC103403121). In order to compare the stability of the expression intensities of these two candidate housekeeping genes based on cycle quantification values, the comparative ΔCt method [124], geNorm [125], NormFinder [126], and BestKeeper [127] statistical methods were used along with RefFinder comprehensive tool [128,129] (Table S5).

Two target genes with a negative logarithmic fold change value (2-oxoglutarate-dependent dioxygenase 19-like, LOC139196321 and triacylglycerol lipase OBL1-like, LOC103431301) and two with a positive value (two-component response regulator ARR5-like, LOC103437756 and (R)-mandelonitrile beta-glucosyltransferase-like, LOC103448619) were selected from RNA-seq gene expression data as candidate target genes.

The sequences were obtained from the National Center for Biotechnology Information (NCBI). SnapGene (v8.0) software was used for the primer design. For evaluation of suitability, potential primers were also checked with PCR Primer Stats (www.bioinformatics.org accessed on 15 September 2025). The conditions used for the primer design were as follows: 18–25 base pairs (bp) of primer length with 40–60% GC content and PCR product length of 150–250 bp. The melting temperature was set between 56 and 62 °C. Using NCBI BLASTn (Basic Local Alignment Sequence Tool for nucleotides), each potential amplicon was tested for homology with other genes. All primers were synthetized by Integrated DNA Technologies (IDT, Leuven, Belgium).

RT-qPCR analysis was performed using the Takyon™ No Rox^®^ SYBR MasterMix dTTP Blue kit (Eurogentec, Searing, Belgium) following the manufacturer’s protocol. A final reaction volume of 20 µL consisted of 2.5 µL of cDNA, 2.0 µL of each oligonucleotide, 10 µL of Takyon™ MasterMix and 3.5 µL of water. The primers (both reverse and forward) were used in a concentration of 0.1 µM. The amplification protocol consisted of three main steps. The first, Takyon™ activation step was set to 95 °C for 3 min, followed by 40 cycles of 95 °C for 10 sec (denaturation), a primer-specific annealing temperature of 54 °C for 20 sec, and an extension step of 72 °C for 30 sec. For each biological replicate, three technical replicates were used (for every treatment group, BA, TOP and NCK).

Relative gene expression was calculated with the 2^(−ΔΔCq)^ method [130] using the mean of three technical replicates for each biological replicate. Using Excel (Microsoft, Redmond, WA, USA), the gene expression logarithmic fold change values (LFC values) were calculated along with standard deviations. The Spearman correlation coefficient between mRNA-seq and RT-qPCR data was calculated using SRplot (https://www.bioinformatics.com.cn/en accessed on 22 September 2025) (Table S5).

## 5. Conclusions

Although the effects of cytokinins in vitro appear to be a well-researched area, this is not the case in the sense that their effects on gene transcription of in vitro shoots are yet unexplored. Investigating the transcriptomic effects of exogenous cytokinin supplementation of the culture medium, namely *meta*-topolin and benzyl adenine, revealed how both cytokinins differentially affected auxin transport and signaling as well as auxin synthesis and cytokinin and gibberellin biosynthesis. These transcriptomic alterations in response to the two different cytokinin treatments that lead to changes in the cytokinin–auxin balance and gibberellin biosynthesis in in vitro apple shoots may contribute to understanding the morphological differences previously observed for the two cytokinins [3]. Further investigations related to the direct measurements of hormone content in in vitro shoots cultured on the BA- or TOP-containing medium may contribute to confirming the findings of this study.

## Figures and Tables

**Table 1:** Total number of differentially expressed genes (DEGs) and number of up (↑)- or down (↓)-regulated DEGs, in BA vs. NCK, TOP vs. NCK, and TOP vs. BA comparisons four weeks after culturing in vitro shoots of apple (*Malus x domestica* Borkh. cv. Húsvéti rozmaring) on shoot multiplication media containing different types of cytokinins (BA or TOP) or no (NCK) cytokinin.

Treatment Comparisons	BA vs. NCK	TOP vs. NCK	TOP vs. BA
Total number of DEGs	2956	468	237
Up-regulated DEGs (↑)	2028	309	178
Down-regulated DEGs (↓)	928	159	59

**Table 2:** Number of significantly up (↑)-, and down (↓)-regulated DEGs related to pathways of metabolism, genetic information processing and cellular processes, based on KEGG mapping (BA: benzyl adenine; CK-free: cytokinin-free; TOP: *meta*-topolin).

Pathways		BA vs. CK-Free	TOP vs. CK-Free	TOP vs. BA
**Metabolism**				
	**Global**			
	Metabolic pathways	↓12 ↑3	↓4 ↑1	
	Biosynthesis of secondary metabolites	↓8 ↑2		
	**Lipid metabolism**			
	Linoleic acid metabolism			↑1
	**Amino acid metabolism**			
	Valine, leucine and isoleucine degradation	↓2		
	**Metabolism of terpenoids and polyketides**			
	Diterpenoid biosynthesis; including Gibberellin biosynthesis			↑1
**Cellular Processes**				
	**Transport and catabolism**			
	Efferocytosis			↑1

## Data Availability

The transcriptomics data has been deposited at the NCBI SRA database (https://www.ncbi.nlm.nih.gov/sra), where it is available under the BioProject identifier PRJNA1358718. Apart from that, all relevant data can be found within the manuscript and its Supplementary Materials.

## Supplementary Materials

The following supporting information can be downloaded at: https://www.mdpi.com/article/10.3390/plants14233691/s1, Table S1: Significantly up- and down-regulated genes (DEGs) at cut-off value of LFC ≥ 1, based on mRNA-seq of 51,804 genes (promoters and coding regions) in comparisons of BA vs. control (HR_BA_vs_HR_NCK_DEG_lfc_1), TOP vs. control (HR_BA_vs_HR_NCK_DEG_lfc_1), and TOP vs. BA (HR_TOP_vs_HR_BA_DEG_lfc_1), respectively, four weeks after various cytokinin supplies applied in the shoot multiplication medium of in vitro apple (cv. Húsvéti rozmaring) shoots; Table S2: GO enrichment for significantly up- and down-regulated biological processes, molecular functions and cellular components in response to BA (HR_BA_vs_HR_NCK), and TOP (HR_TOP_vs_HR_NCK) supply compared to control, respectively; Table S3: Affected metabolic pathways and cellular processes based on KEGG mapping in comparisons of BA vs. control (HR_BA_vs_HR_NCK), TOP vs. control (HR_TOP_vs_HR_NCK), and TOP vs. BA (HR_TOP_vs._HR_BA), respectively, four weeks after various cytokinin supplies applied in the shoot multiplication medium of in vitro apple (cv. Húsvéti rozmaring) shoots; Table S4: List of enzymes related to the significantly up- and down-regulated DEGs based on KEGG mapping in comparisons of BA vs. control, TOP vs. control, and TOP vs. BA, respectively; Table S5: Validation of mRNA-seq data by RT-qPCR. The associated supporting data is presented across three distinct worksheets within a single file. The first worksheet provides a comprehensive list of all primer sequences used and their associated data. The second worksheet details the evaluation process for identifying and selecting suitable housekeeping genes. Finally, the third worksheet contains both the raw PCR results and a thorough analysis of those findings; Figure S1: Heat maps showing expression intensity of the top 100 significantly up- or down-regulated DEGs in various cytokinin supply (control (NCK), BA and TOP, respectively). Heat maps generated by ComplexHeatmap R package; Figure S2: Volcano plots on total number of expressed genes in in vitro apple shoots in comparisons between (**A**) BA vs. control (NCK), (**B**) TOP vs. control, and (**C**) TOP vs. BA, and total number of significantly differentially expressed, up- and down-regulated genes in the same comparisons, four weeks after in vitro cultivation. Volcano plots were generated by ggplot2 R package; *x*-axis representing the log2(Fold Change) and *y*-axis the adjusted *p*-values of the genes. The blue dots indicate down-regulated gene expression, the red dots indicate up-regulated gene expression, while the gray dots indicate gene expression without significant differences, in each comparison; Figure S3: GO enrichment dot plots based on significantly up- and down-regulated genes enriched in biological processes, molecular functions and cellular components. Dot plots were generated with ClusterProfiler R package (BA: benzyl adenine; TOP: meta-topolin; NCK: control, cytokinin-free medium); Figure S4: KEGG maps of affected metabolic pathways and cellular processes in comparisons of BA vs. NCK (control), TOP vs. NCK (control), and TOP vs. BA, respectively, four weeks after various cytokinin supplies applied in the shoot multiplication medium of in vitro apple (cv. Húsvéti rozmaring) shoots. Figures were generated with pathview R package.
