# Supplementary material for: Differential Alteration of Gene Expression by Benzyl Adenine and meta-Topolin in In Vitro Apple Shoots
Source: Plants (Basel). 2025 Dec 4;14(23):3691. doi: 10.3390/plants14233691 (PMC12694410; doi:10.3390/plants14233691)
Supplement: Supplementary file 1 [file plants-14-03691-s001.zip › Figure S3.pdf]

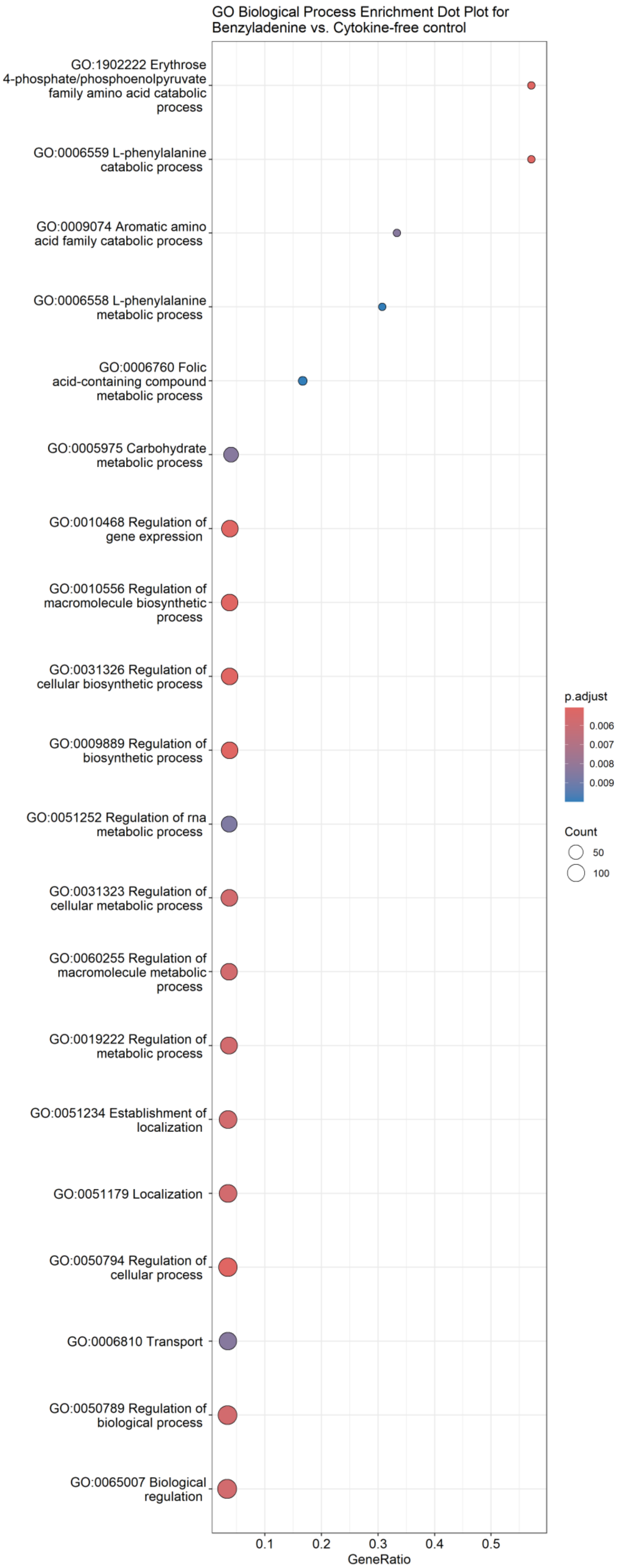

GO Biological Process Enrichment Dot Plot for Benzyladenine vs. Cytokine-free control
GO:1902222 Erythrose 4-phosphate/phosphoenolpyruvate family amino acid catabolic process
GO:0006559 L-phenylalanine catabolic process
GO:0009074 Aromatic amino acid family catabolic process
GO:0006558 L-phenylalanine metabolic process
GO:0006760 Folic acid-containing compound metabolic process
GO:0005975 Carbohydrate metabolic process
GO:0010468 Regulation of gene expression
GO:0010556 Regulation of macromolecule biosynthetic process
GO:0031326 Regulation of cellular biosynthetic process
GO:0009889 Regulation of biosynthetic process
GO:0051252 Regulation of rna metabolic process
GO:0031323 Regulation of cellular metabolic process
GO:0060255 Regulation of macromolecule metabolic process
GO:0019222 Regulation of metabolic process
GO:0051234 Establishment of localization
GO:0051179 Localization
GO:0050794 Regulation of cellular process
GO:0006810 Transport
GO:0050789 Regulation of biological process
GO:0065007 Biological regulation
0.1
0.2
0.3
0.4
0.5
GeneRatio
p.adjust
0.006
0.007
0.008
0.009
Count
50
100

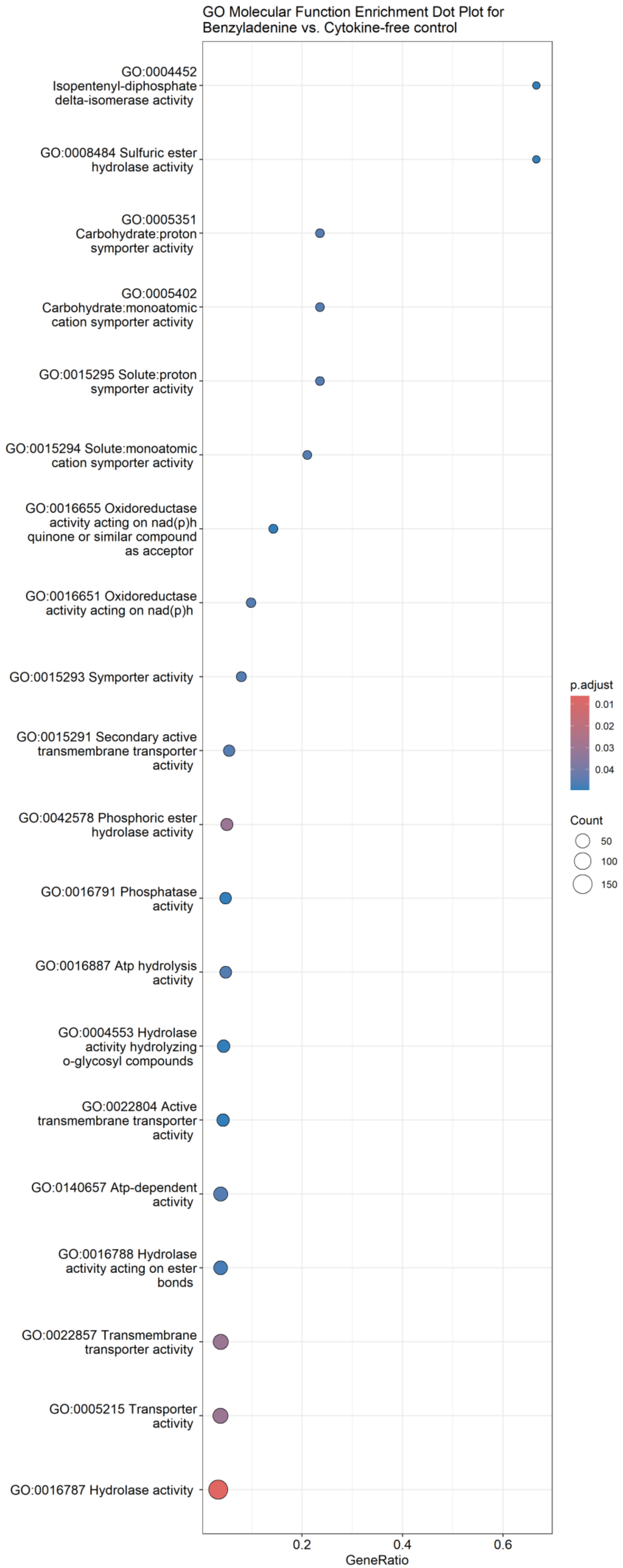

GO Molecular Function Enrichment Dot Plot for
Benzyladenine vs. Cytokine-free control
GO:0004452 Isopentenyl-diphosphate delta-isomerase activity
GO:0008484 Sulfuric ester hydrolase activity
GO:0005351 Carbohydrate:proton symporter activity
GO:0005402 Carbohydrate:monoatomic cation symporter activity
GO:0015295 Solute:proton symporter activity
GO:0015294 Solute:monoatomic cation symporter activity
GO:0016655 Oxidoreductase activity acting on nad(p)h quinone or similar compound as acceptor
GO:0016651 Oxidoreductase activity acting on nad(p)h
GO:0015293 Symporter activity
GO:0015291 Secondary active transmembrane transporter activity
GO:0042578 Phosphoric ester hydrolase activity
GO:0016791 Phosphatase activity
GO:0016887 Atp hydrolysis activity
GO:0004553 Hydrolase activity hydrolyzing o-glycosyl compounds
GO:0022804 Active transmembrane transporter activity
GO:0140657 Atp-dependent activity
GO:0016788 Hydrolase activity acting on ester bonds
GO:0022857 Transmembrane transporter activity
GO:0005215 Transporter activity
GO:0016787 Hydrolase activity
0.2
0.4
0.6
GeneRatio
p.adjust
0.01
0.02
0.03
0.04
Count
50
100
150

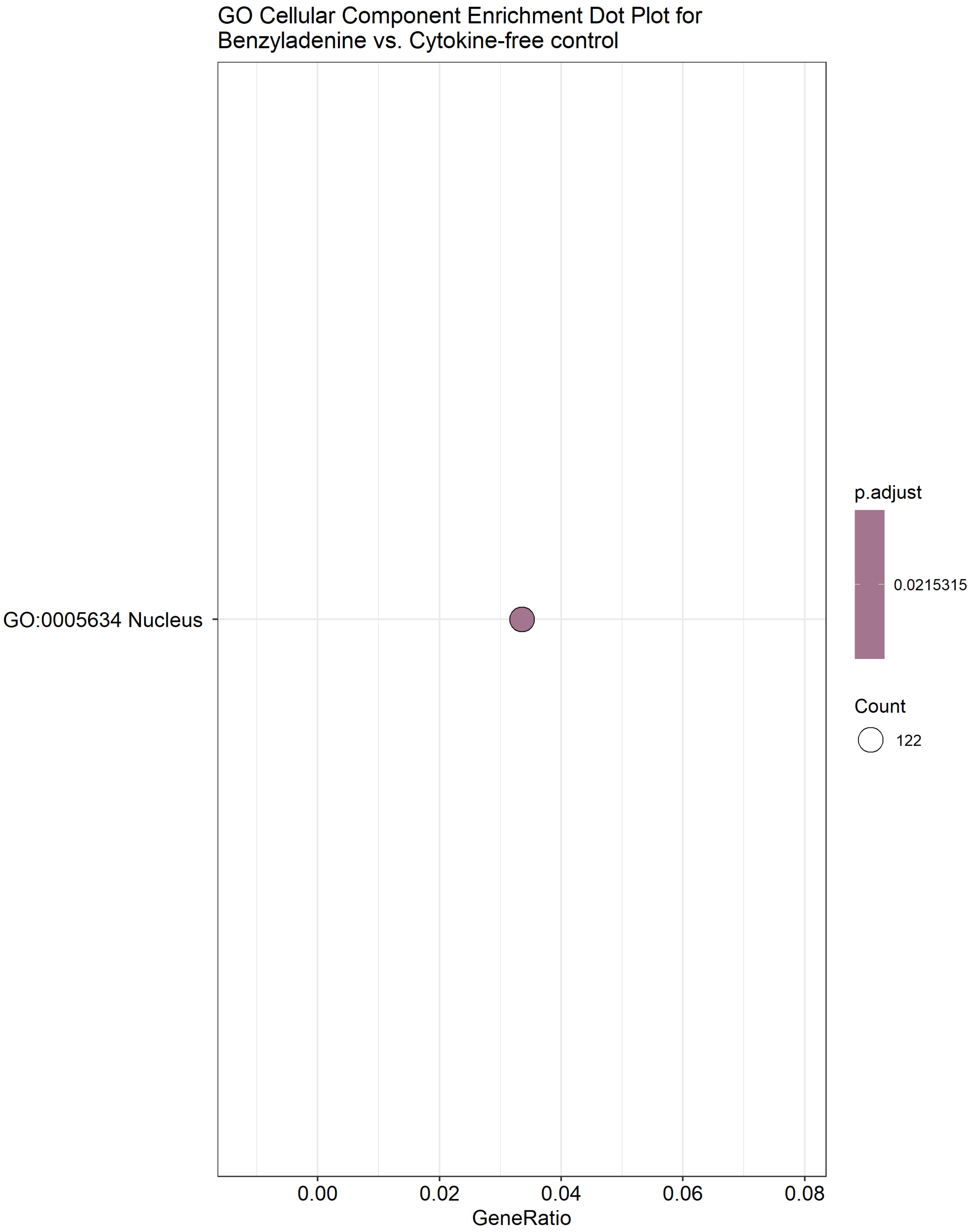
GO Cellular Component Enrichment Dot Plot for
Benzyladenine vs. Cytokine-free control
GO:0005634 Nucleus
0.00
0.02
0.04
0.06
0.08
GeneRatio
p.adjust
0.0215315
Count
122

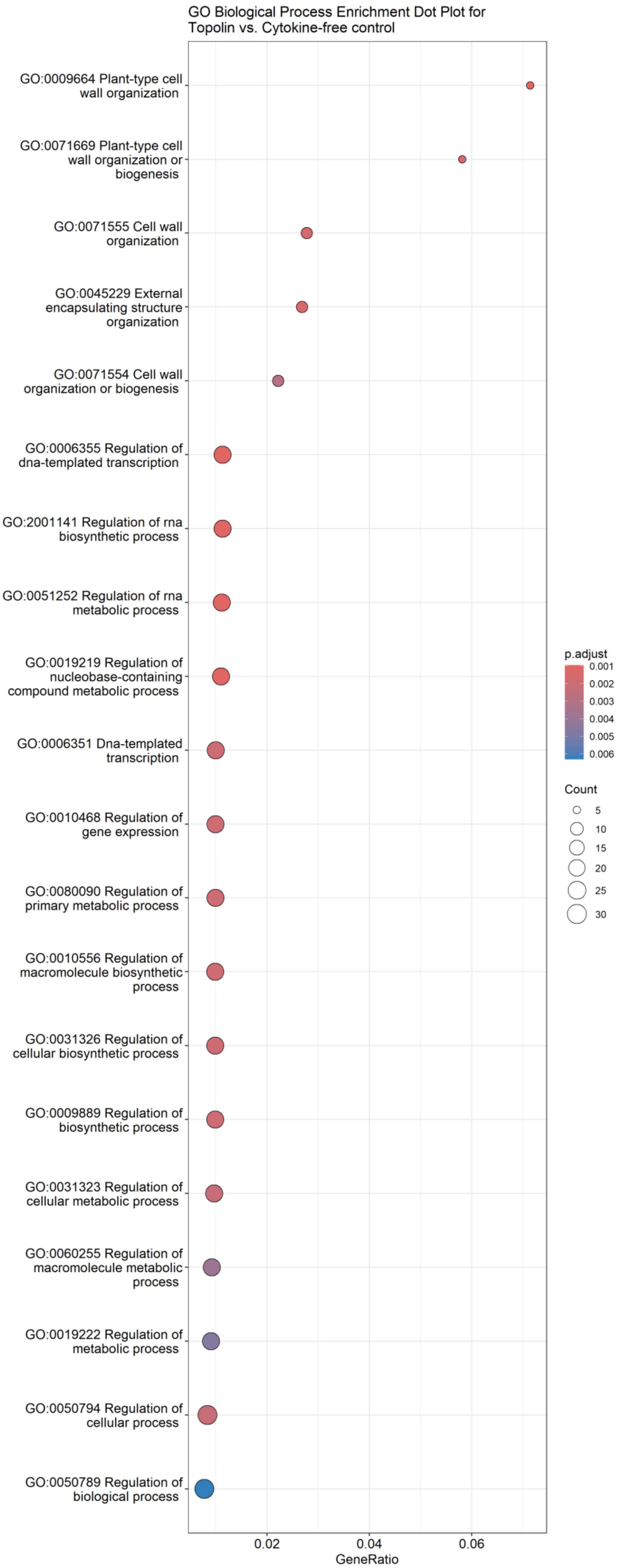
GO Biological Process Enrichment Dot Plot for
Topolin vs. Cytokine-free control
GO:0009664 Plant-type cell wall organization
GO:0071669 Plant-type cell wall organization or biogenesis
GO:0071555 Cell wall organization
GO:0045229 External encapsulating structure organization
GO:0071554 Cell wall organization or biogenesis
GO:0006355 Regulation of dna-templated transcription
GO:2001141 Regulation of rna biosynthetic process
GO:0051252 Regulation of rna metabolic process
GO:0019219 Regulation of nucleobase-containing compound metabolic process
GO:0006351 Dna-templated transcription
GO:0010468 Regulation of gene expression
GO:0080090 Regulation of primary metabolic process
GO:0010556 Regulation of macromolecule biosynthetic process
GO:0031326 Regulation of cellular biosynthetic process
GO:0009889 Regulation of biosynthetic process
GO:0031323 Regulation of cellular metabolic process
GO:0060255 Regulation of macromolecule metabolic process
GO:0019222 Regulation of metabolic process
GO:0050794 Regulation of cellular process
GO:0050789 Regulation of biological process
0.02
0.04
0.06
GeneRatio
p.adjust
0.001
0.002
0.003
0.004
0.005
0.006
Count
5
10
15
20
25
30

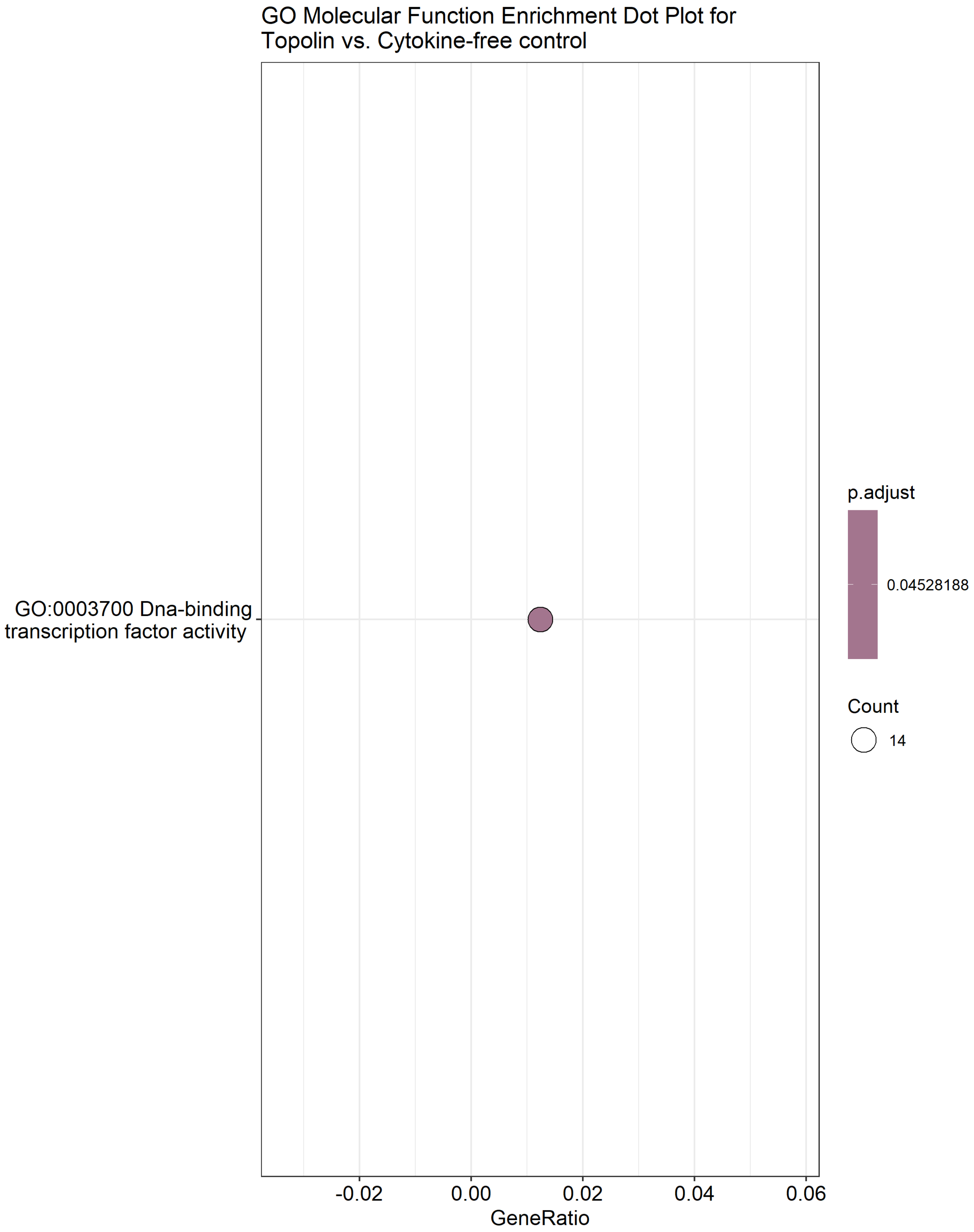
GO Molecular Function Enrichment Dot Plot for
Topolin vs. Cytokine-free control
GO:0003700 Dna-binding
transcription factor activity
-0.02
0.00
0.02
0.04
0.06
GeneRatio
p.adjust
0.04528188
Count
14

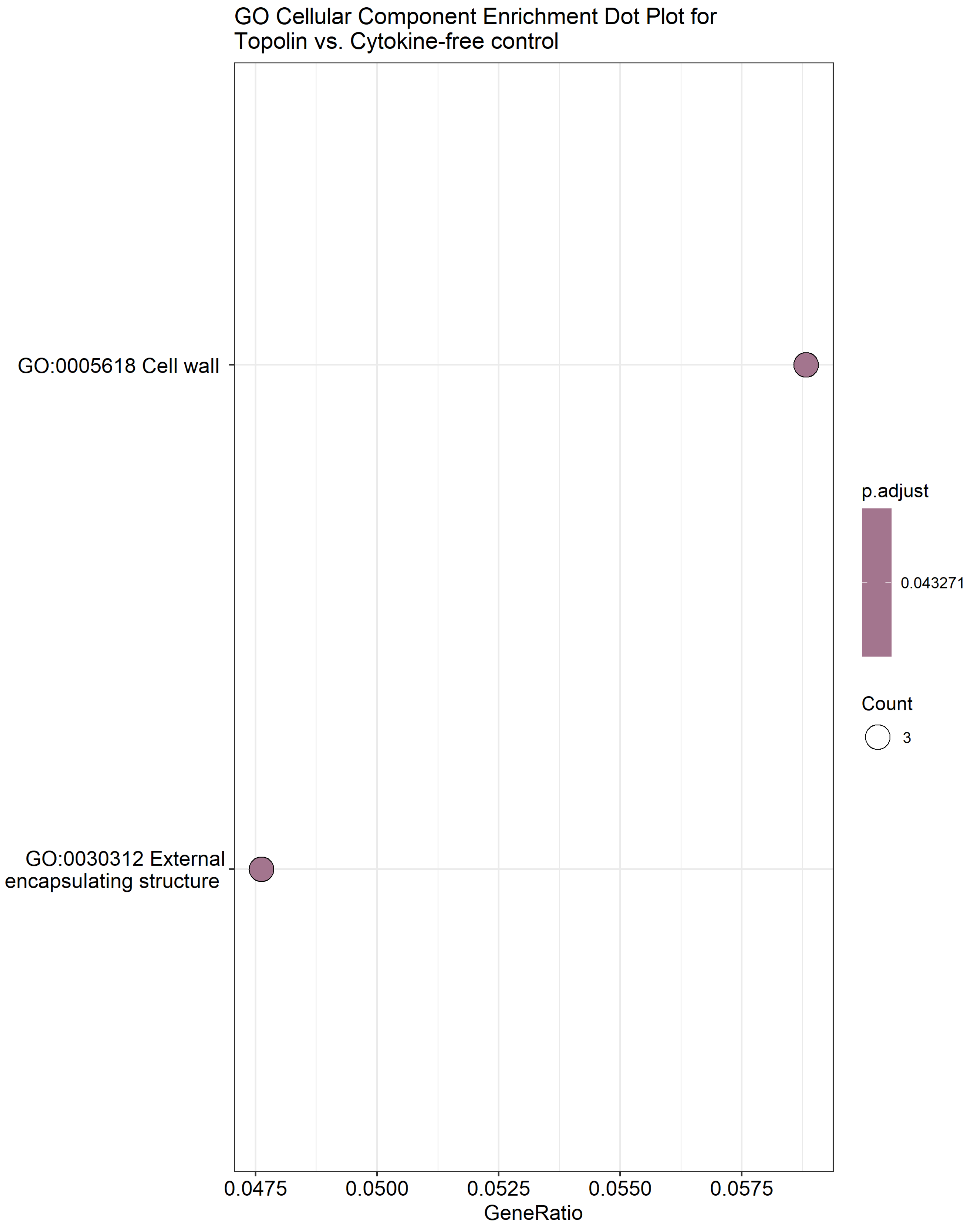
GO Cellular Component Enrichment Dot Plot for
Topolin vs. Cytokine-free control
GO:0005618 Cell wall
GO:0030312 External
encapsulating structure
0.0475
0.0500
0.0525
0.0550
0.0575
GeneRatio
p.adjust
0.043271
Count
3
