# Supplementary material for: Differential Alteration of Gene Expression by Benzyl Adenine and meta-Topolin in In Vitro Apple Shoots
Source: Plants (Basel). 2025 Dec 4;14(23):3691. doi: 10.3390/plants14233691 (PMC12694410; doi:10.3390/plants14233691)
Supplement: Supplementary file 1 [file plants-14-03691-s001.zip › Figure S4.pdf]

BA vs. NCK

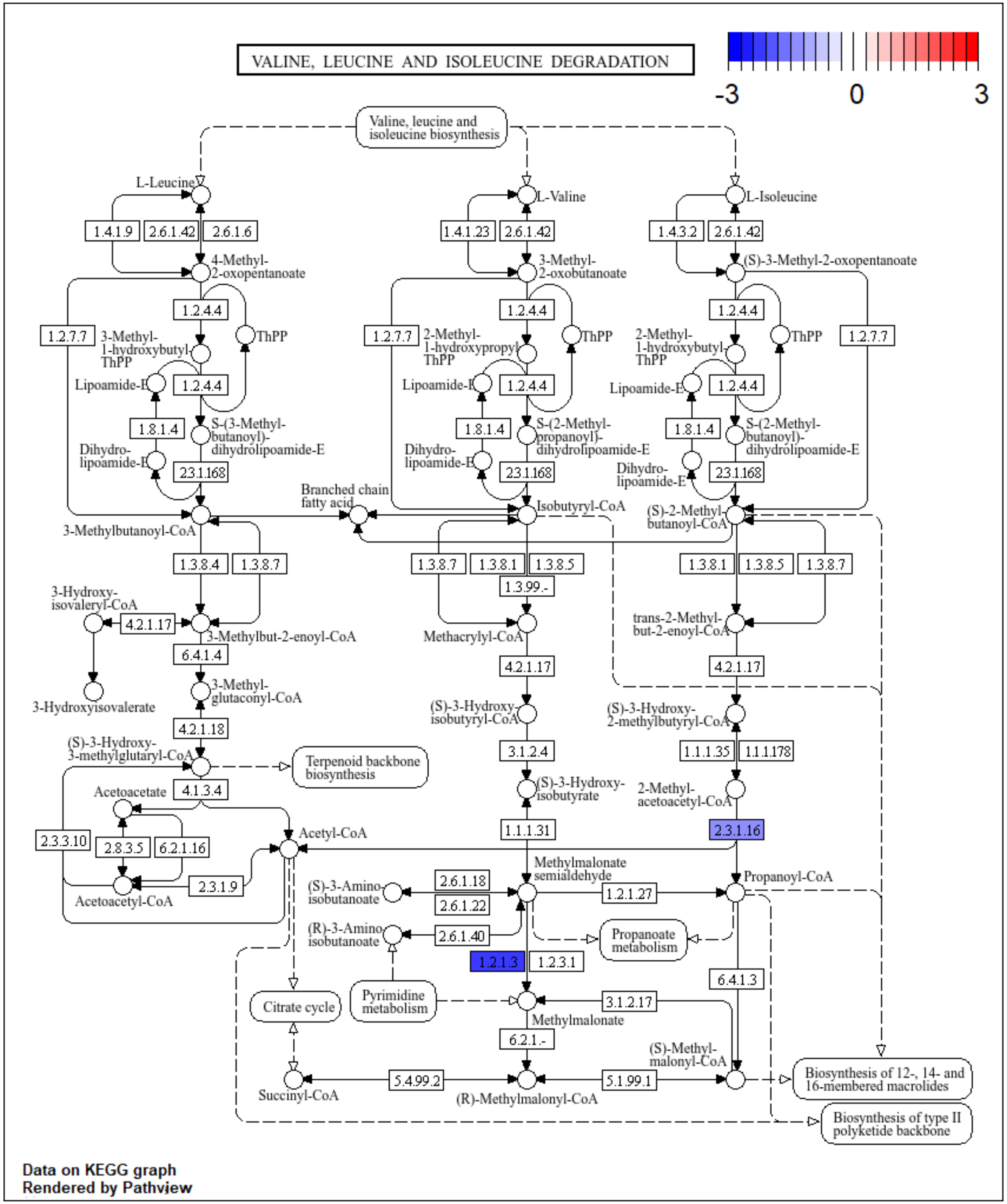

VALINE, LEUCINE AND ISOLEUCINE DEGRADATION
-3
0
3
Valine, leucine and isoleucine biosynthesis
L-Leucine
L-Valine
L-Isoleucine
1.4.1.9
2.6.1.42
2.6.1.6
1.4.1.23
1.4.3.2
4-Methyl-2-oxopentanoate
3-Methyl-2-oxobutanoate
(S)-3-Methyl-2-oxopentanoate
1.2.4.4
1.2.7.7
ThPP
3-Methyl-1-hydroxybutyl-ThPP
2-Methyl-1-hydroxypropyl-ThPP
2-Methyl-1-hydroxybutyl-ThPP
Lipoamide-E
1.8.1.4
S-(3-Methylbutanoyl)-dihydrolipoamide-E
S-(2-Methylpropanoyl)-dihydrolipoamide-E
S-(2-Methylbutanoyl)-dihydrolipoamide-E
Dihydrolipoamide-E
2.3.1.168
Branched chain fatty acid
3-Methylbutanoyl-CoA
Isobutyryl-CoA
(S)-2-Methylbutanoyl-CoA
1.3.8.4
1.3.8.7
1.3.8.1
1.3.8.5
1.3.99.-
3-Hydroxyisovaleryl-CoA
4.2.1.17
3-Methylbut-2-enoyl-CoA
Methacrylyl-CoA
trans-2-Methylbut-2-enoyl-CoA
6.4.1.4
3-Hydroxyisovalerate
3-Methylglutaconyl-CoA
4.2.1.18
(S)-3-Hydroxyisobutyryl-CoA
(S)-3-Hydroxy-2-methylbutyryl-CoA
(S)-3-Hydroxy-3-methylglutaryl-CoA
Terpenoid backbone biosynthesis
3.1.2.4
1.1.1.35
1.1.1.178
Acetoacetate
4.1.3.4
(S)-3-Hydroxyisobutyrate
2-Methylacetoacetyl-CoA
Acetyl-CoA
1.1.1.31
2.3.1.16
2.3.3.10
2.8.3.5
6.2.1.16
Methylmalonate semialdehyde
Propanoyl-CoA
2.3.1.9
(S)-3-Aminoisobutanoate
2.6.1.18
1.2.1.27
Acetoacetyl-CoA
2.6.1.22
(R)-3-Aminoisobutanoate
2.6.1.40
Propanoate metabolism
1.2.1.3
1.2.3.1
6.4.1.3
Citrate cycle
Pyrimidine metabolism
3.1.2.17
Methylmalonate
6.2.1.-
(S)-Methylmalonyl-CoA
Biosynthesis of 12-, 14- and 16-membered macrolides
Succinyl-CoA
5.4.99.2
(R)-Methylmalonyl-CoA
5.1.99.1
Biosynthesis of type II polyketide backbone
Data on KEGG graph
Rendered by Pathview

BA vs. NCK

METABOLIC PATHWAYS

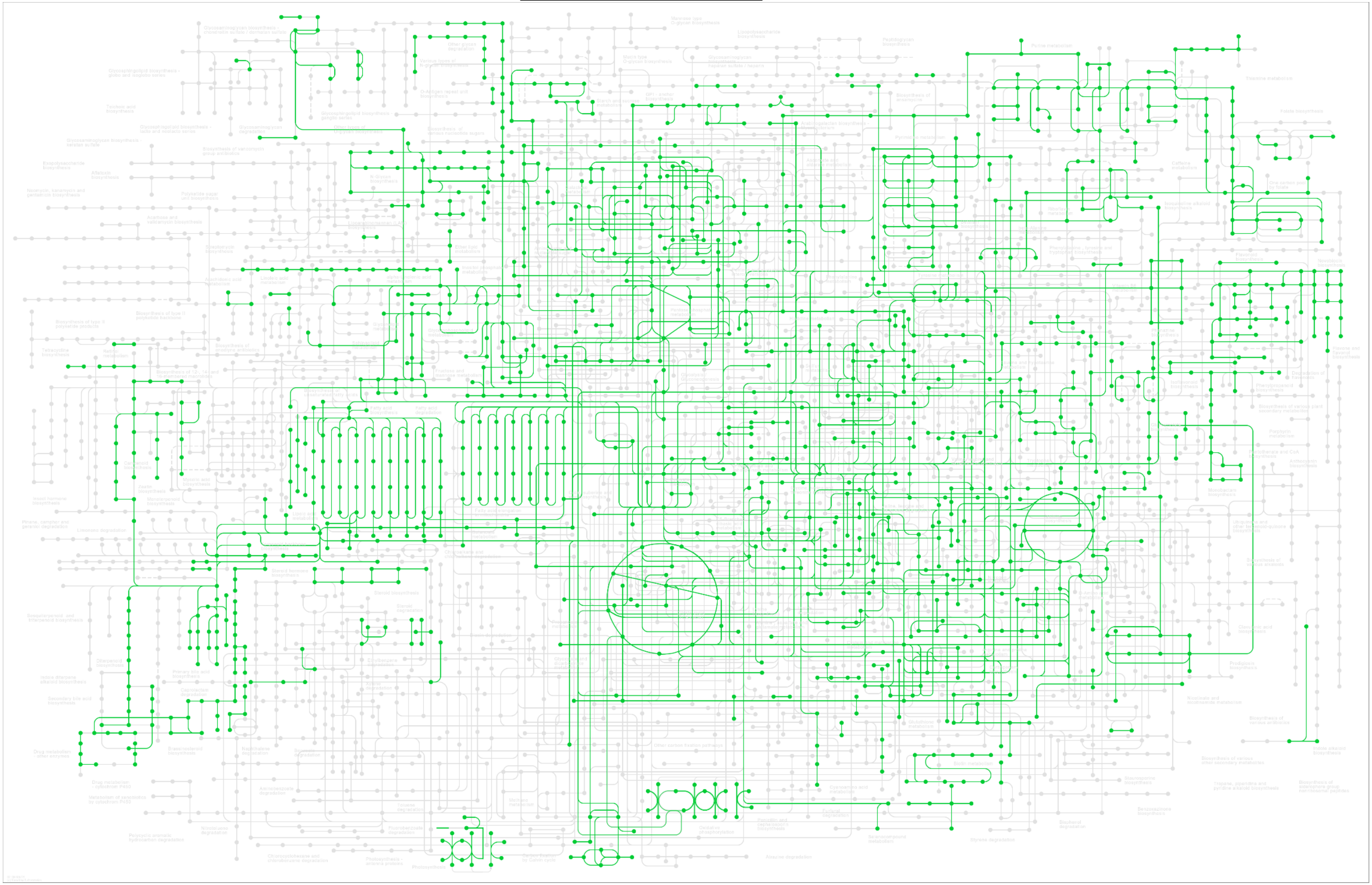

BA vs. NCK

BIOSYNTHESIS OF SECONDARY METABOLITES

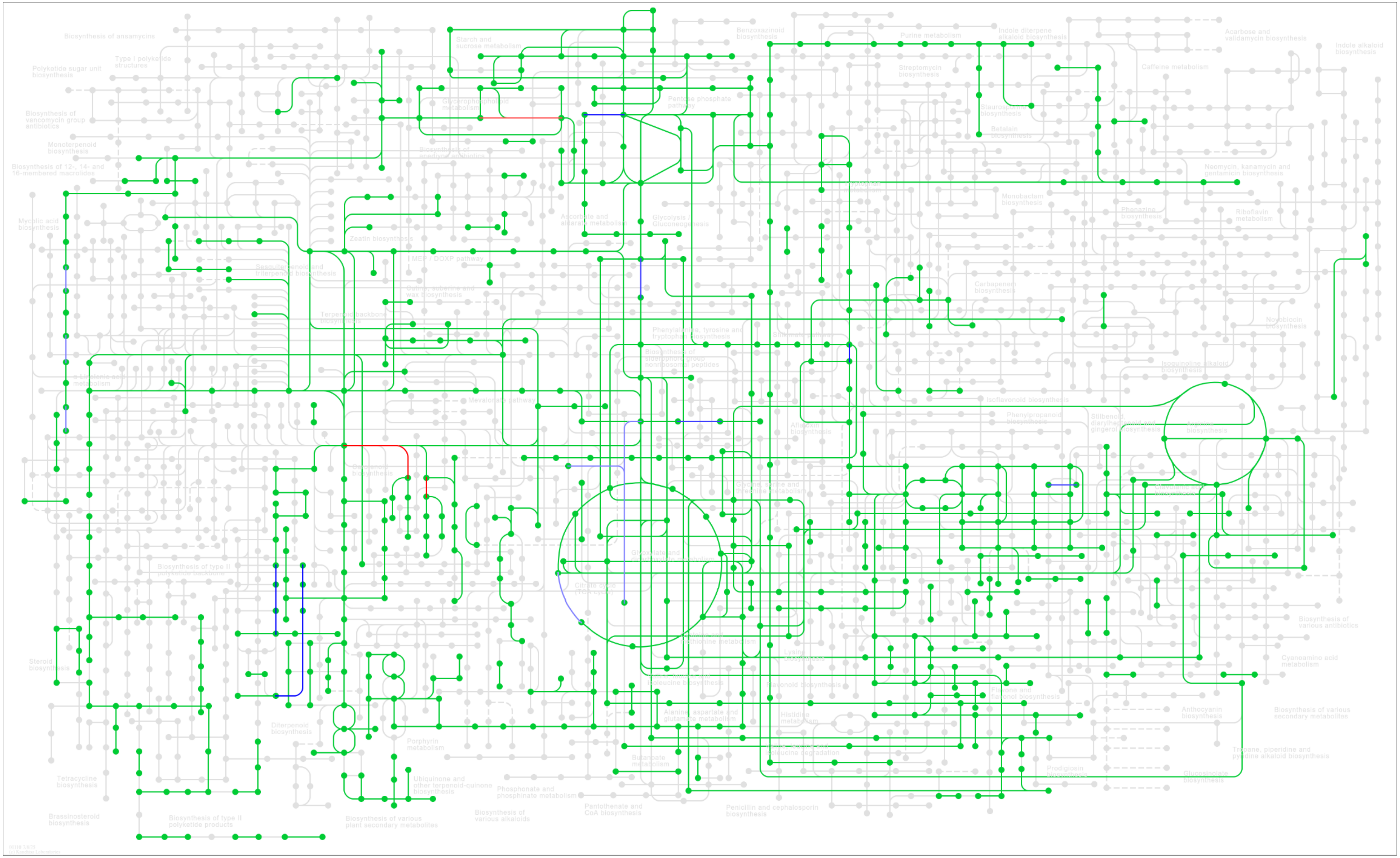

TOP vs. NCK

METABOLIC PATHWAYS

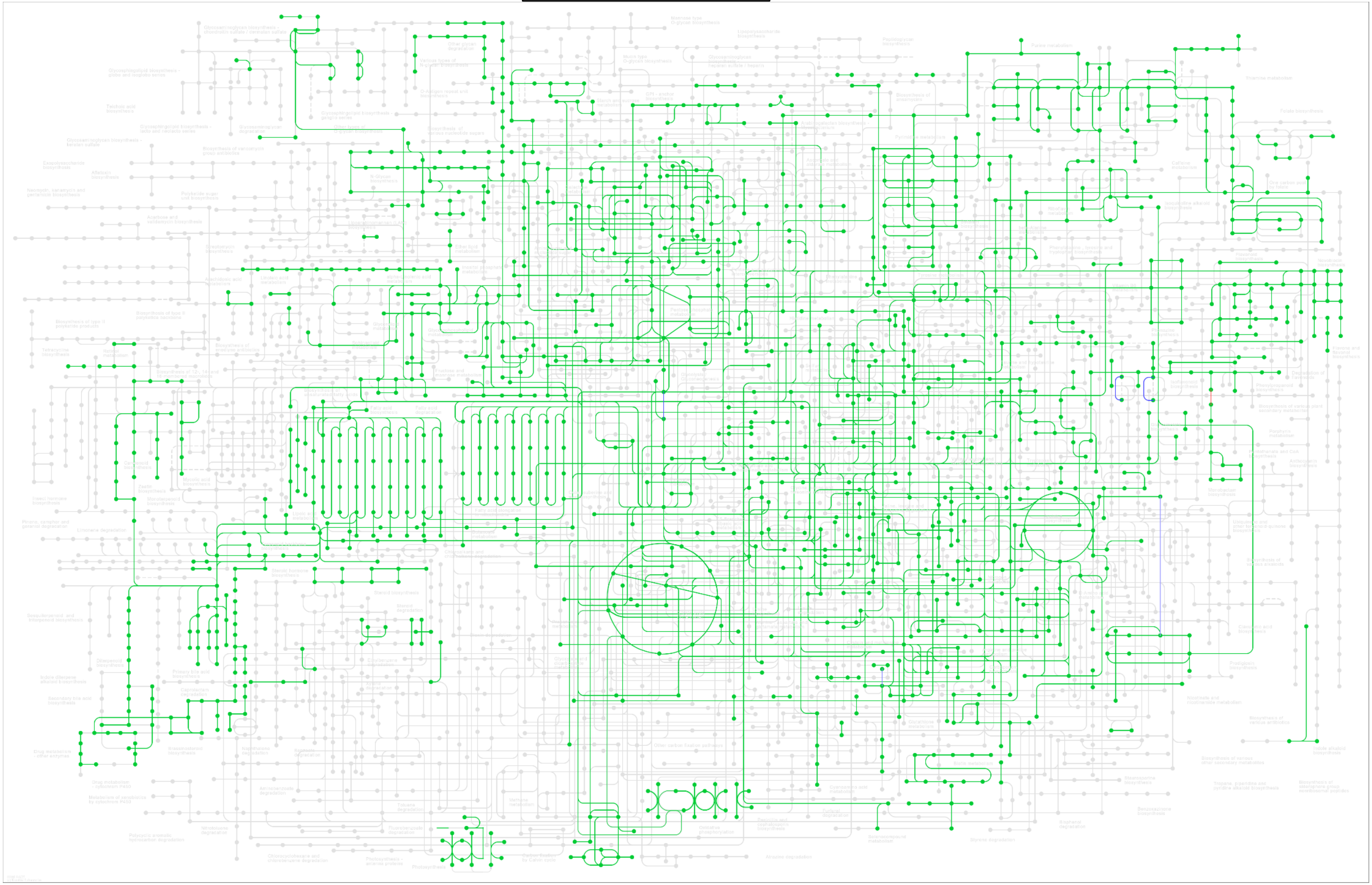

TOP vs. BA

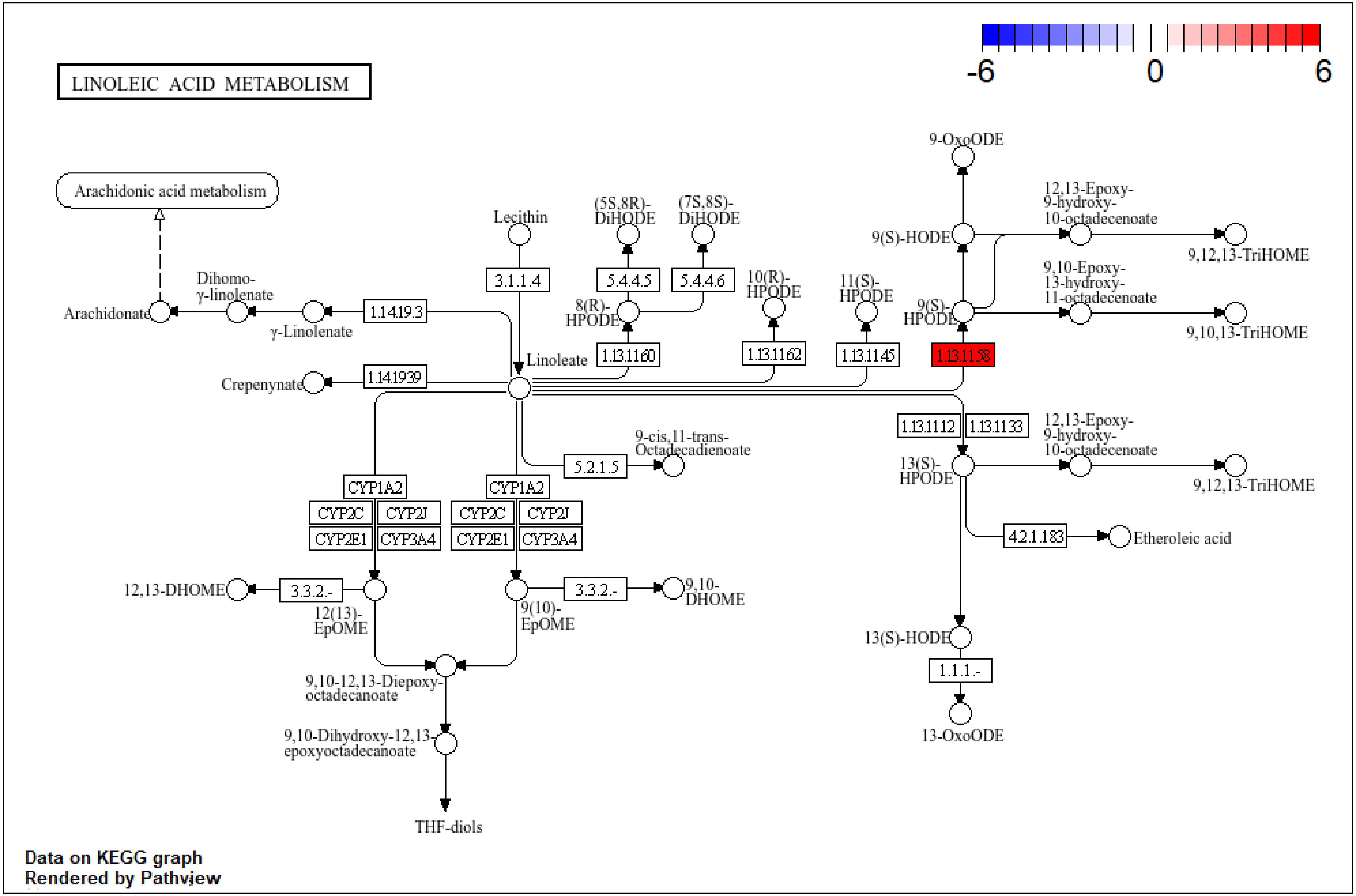

-6
0
6
LINOLEIC ACID METABOLISM
Arachidonic acid metabolism
Arachidonate
Dihomo-
γ-linolenate
γ-Linolenate
1.14.19.3
Lecithin
3.1.1.4
(5S,8R)-
DiHODE
(7S,8S)-
DiHODE
5.4.4.5
5.4.4.6
8(R)-
HPODE
10(R)-
HPODE
11(S)-
HPODE
9-OxoODE
9(S)-HODE
9(S)-
HPODE
12,13-Epoxy-
9-hydroxy-
10-octadecenoate
9,12,13-TriHOME
9,10-Epoxy-
13-hydroxy-
11-octadecenoate
9,10,13-TriHOME
Crepenynate
1.14.19.39
Linoleate
1.13.11.60
1.13.11.62
1.13.11.45
1.13.11.58
1.13.11.12
1.13.11.33
9-cis,11-trans-
Octadecadienoate
5.2.1.5
13(S)-
HPODE
12,13-Epoxy-
9-hydroxy-
10-octadecenoate
9,12,13-TriHOME
4.2.1.183
Etheroleic acid
CYP1A2
CYP2C
CYP2J
CYP2E1
CYP3A4
CYP1A2
CYP2C
CYP2J
CYP2E1
CYP3A4
12,13-DHOME
3.3.2.-
12(13)-
EpOME
9(10)-
EpOME
3.3.2.-
9,10-
DHOME
9,10-12,13-Diepoxy-
octadecanoate
9,10-Dihydroxy-12,13-
epoxyoctadecanoate
THF-diols
13(S)-HODE
1.1.1.-
13-OxoODE
Data on KEGG graph
Rendered by Pathview

TOP vs. BA

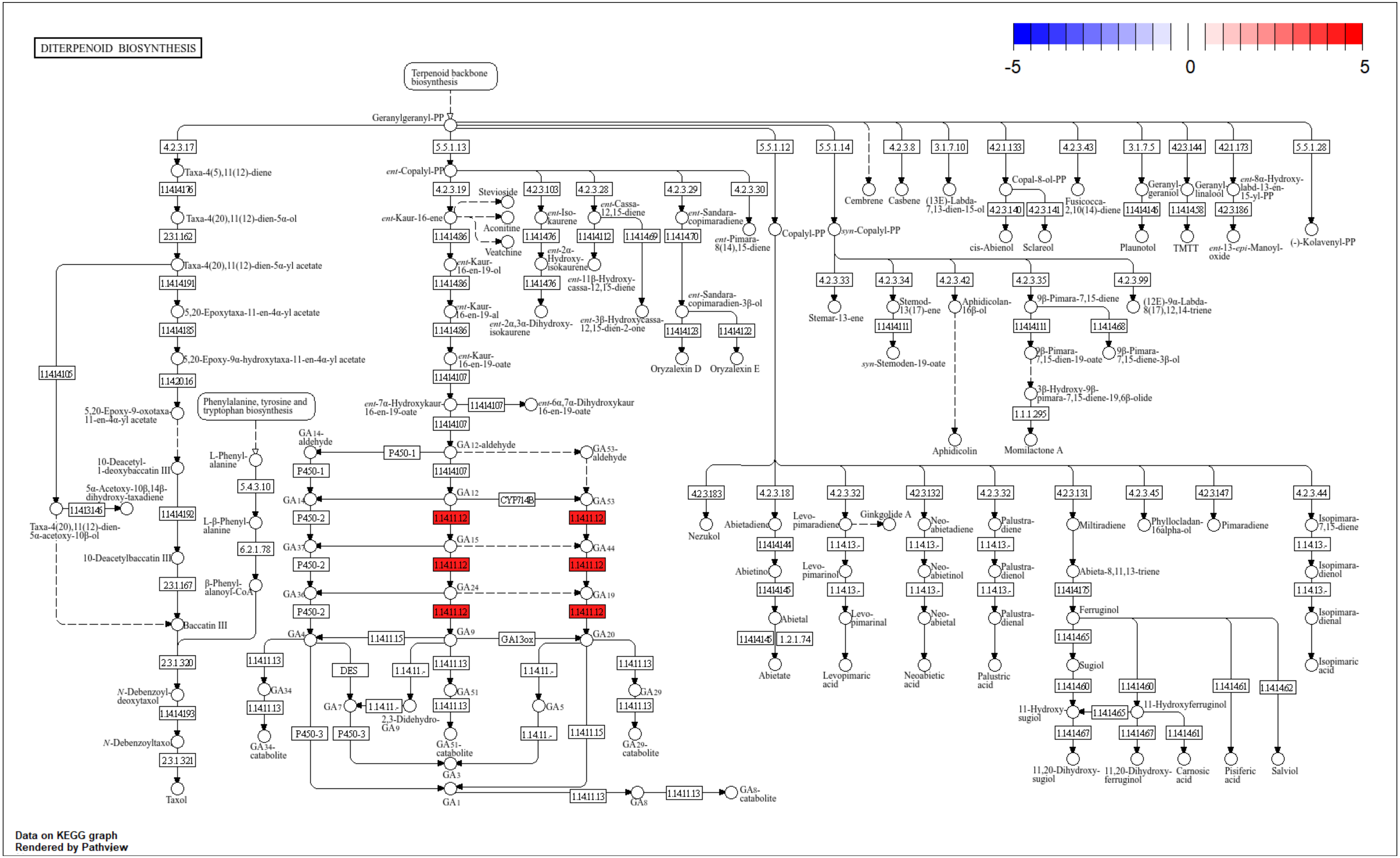
DITERPENOID BIOSYNTHESIS
-5
0
5
Terpenoid backbone biosynthesis
Geranylgeranyl-PP
ent-Copalyl-PP
ent-Kaur-16-ene
Taxa-4(5),11(12)-diene
Taxa-4(20),11(12)-dien-5α-ol
Taxa-4(20),11(12)-dien-5α-yl acetate
Baccatin III
Taxol
Phenylalanine, tyrosine and tryptophan biosynthesis
GA12-aldehyde
GA12
GA15
GA24
GA9
GA20
GA4
GA1
GA3
GA53
GA44
GA19
GA8
CYP714B
GA13ox
Copalyl-PP
syn-Copalyl-PP
Cembrene
Casbene
Abietadiene
Abietic acid
Miltiradiene
Ferruginol
Sugiol
Carnosic acid
Momilactone A
Aphidicolin
Oryzalexin D
Oryzalexin E
Data on KEGG graph
Rendered by Pathview

TOP vs. BA

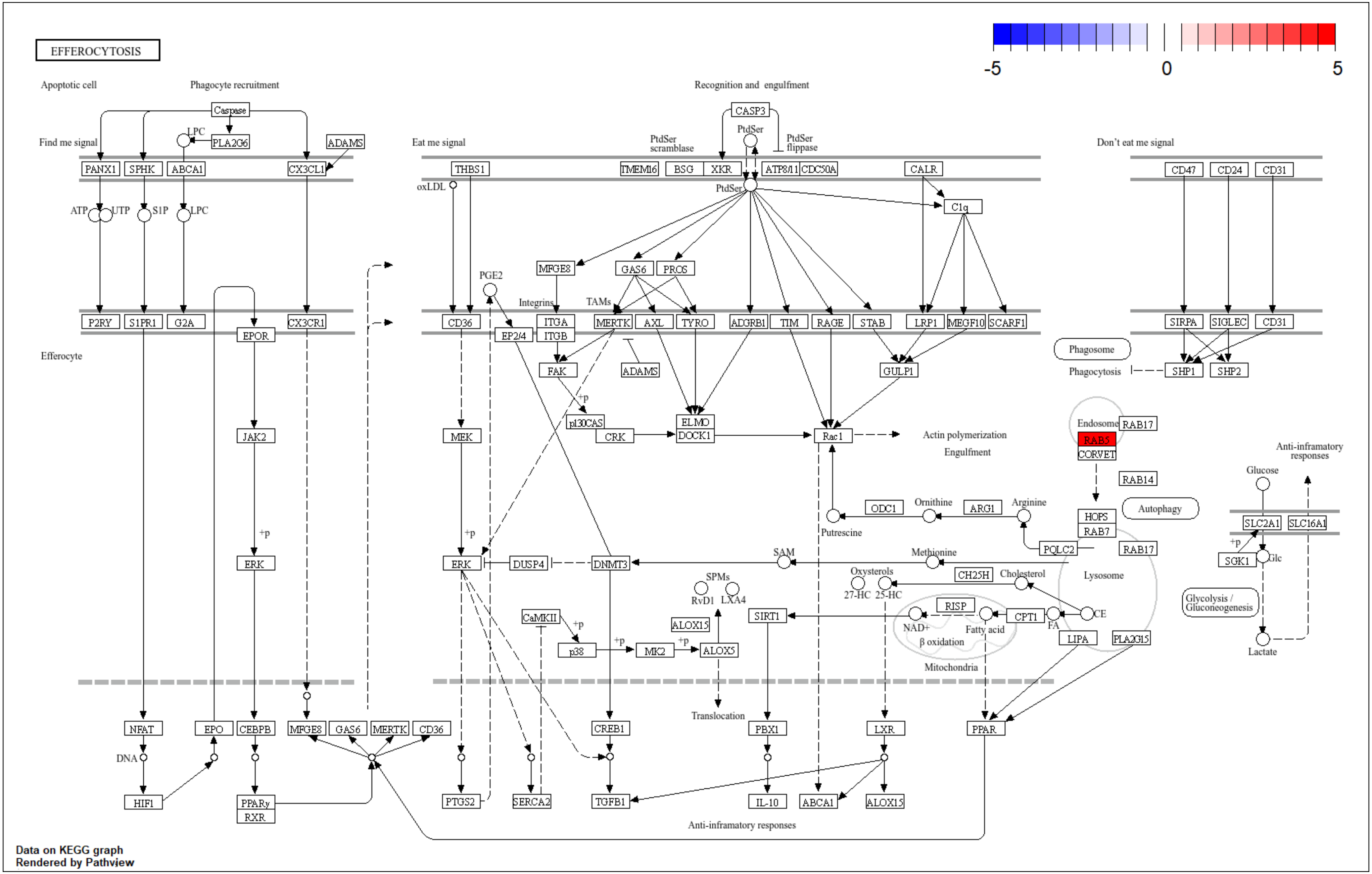

Data on KEGG graph
Rendered by Pathview
