# Supplementary material for: Differential Alteration of Gene Expression by Benzyl Adenine and meta-Topolin in In Vitro Apple Shoots
Source: Plants (Basel). 2025 Dec 4;14(23):3691. doi: 10.3390/plants14233691 (PMC12694410; doi:10.3390/plants14233691)
Supplement: Supplementary file 1 [file plants-14-03691-s001.zip › Table S4.pdf]

Supplementary Table S5. DEGs-related enzymes

Gene ID	Enzyme name	EC number	BA vs. NCK	TOP vs. NCK	TOP vs. BA
LOC103430080	V-type proton ATPase subunit E1 (ATP6V1E1)	EC 3.6.3.14	↑		
LOC103453843	homogentisate phytyltransferase 1, chloroplastic-like (HPT1)	EC 2.5.1.115	↑		
LOC103455409	1,2-diacylglycerol kinase 1-like ATP-dependent (DGK1)	EC 2.7.1.107	↑		
LOC103437475	magnesium protoporphyrin IX methyltransferase (ChlM)	EC 2.1.1.11		↑	
LOC103450936	probable linoleate 9s-lipoxygenase 5 (LOX5)	EC 1.13.11.58			↑
LOC103426352	ras-related protein (RABF1)	N/A			↑
LOC103439897	gibberellin 20 oxidase 2-like (GA20ox2-like)	EC 1.14.11.-	↓		↑
LOC103401166	succinate dehydrogenase (ubiquinone) flavoprotein subunit, mitochondrial (SDHA)	EC 1.3.5.1	↓		
LOC103402682	mannose 6-phosphate isomerase 1-like (PMI)	EC 5.3.1.8	↓		
LOC103404478	chorismate mutase 1 (CM1)	EC 5.4.99.5	↓		
LOC103421546	proline dehydrogenase 2 (PRODH2)	EC 1.5.5.2	↓		
LOC103437735	aldehyde dehydrogenase family 2 member B4, mitochondrial (ALDH2B4)	EC 1.2.1.3	↓		
LOC103443506	3-ketoacyl CoA thiolase, peroxisomal (KAT)	EC 2.3.1.16	↓		
LOC103408692	photosystem I reaction center subunit XI, choloplastic-like (PsaL)	EC 1.97.1.12	↓		
LOC103432314	glutathione S-transferase f12-like/ (GST)	EC 2.5.1.18	↓		
LOC103450793	2,3-bisphospoglycerate-independent phosphoglycerate mutase (iPGAM)	EC 5.4.2.12	↓	↓	

LOC103443500	glutathione reductase, cytosolic (GR)	EC 1.8.1.7	↓	↓	
LOC103402607	quinolinate phosphoribosyltransferase (QRTP)	EC 2.4.2.19	↓	↓	
LOC103434645	NAD(P)H dehydrogenase (quinone) FQR1-LIKE	EC 1.6.5.2		↓	
