# Supplementary figures and images for: Differential Alteration of Gene Expression by Benzyl Adenine and meta-Topolin in In Vitro Apple Shoots
Source: Plants (Basel). 2025 Dec 4;14(23):3691. doi: 10.3390/plants14233691 (PMC12694410; doi:10.3390/plants14233691)

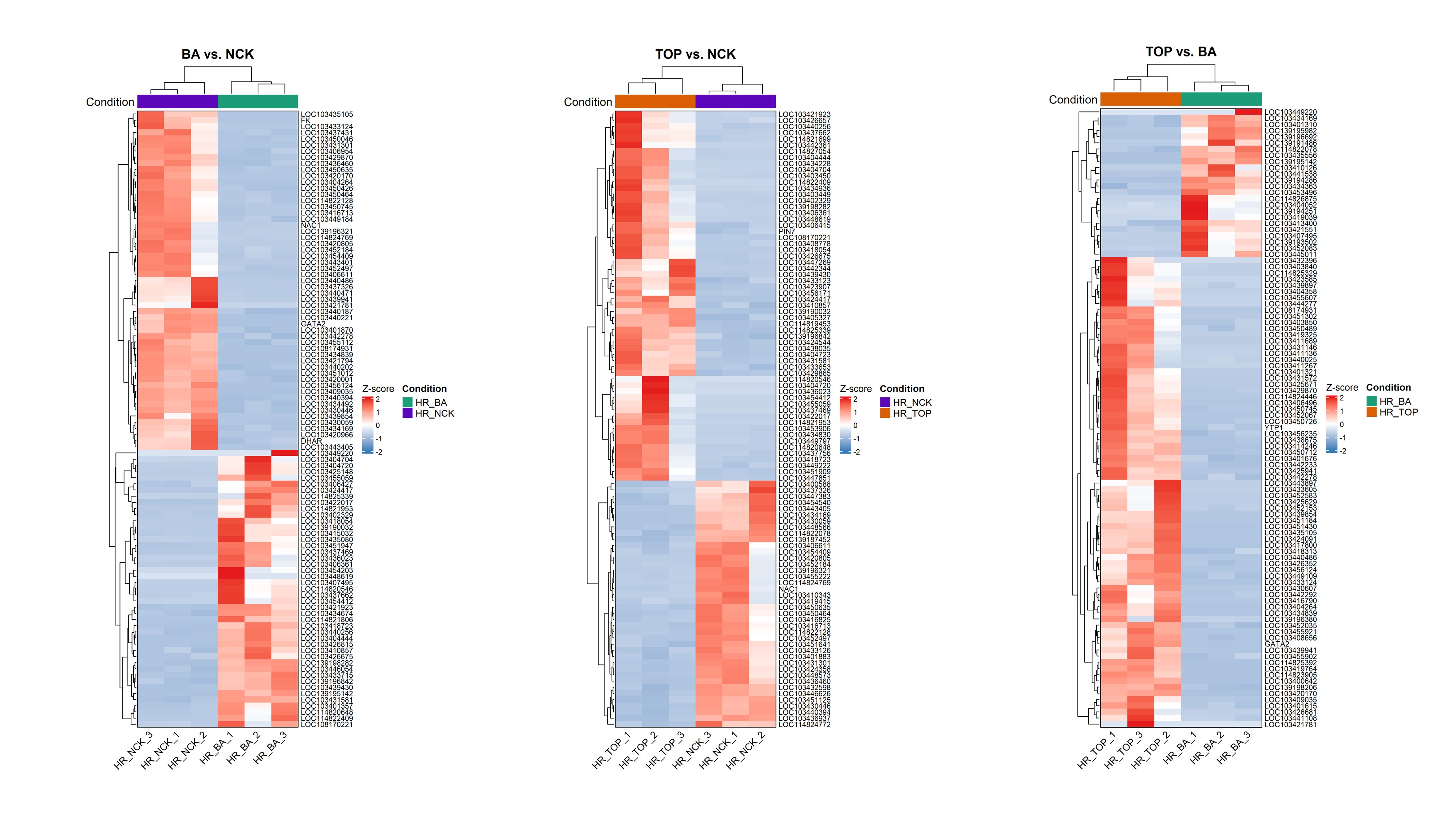

Supplement: Supplementary file 1 [file plants-14-03691-s001.zip › Figure S1.jpg]

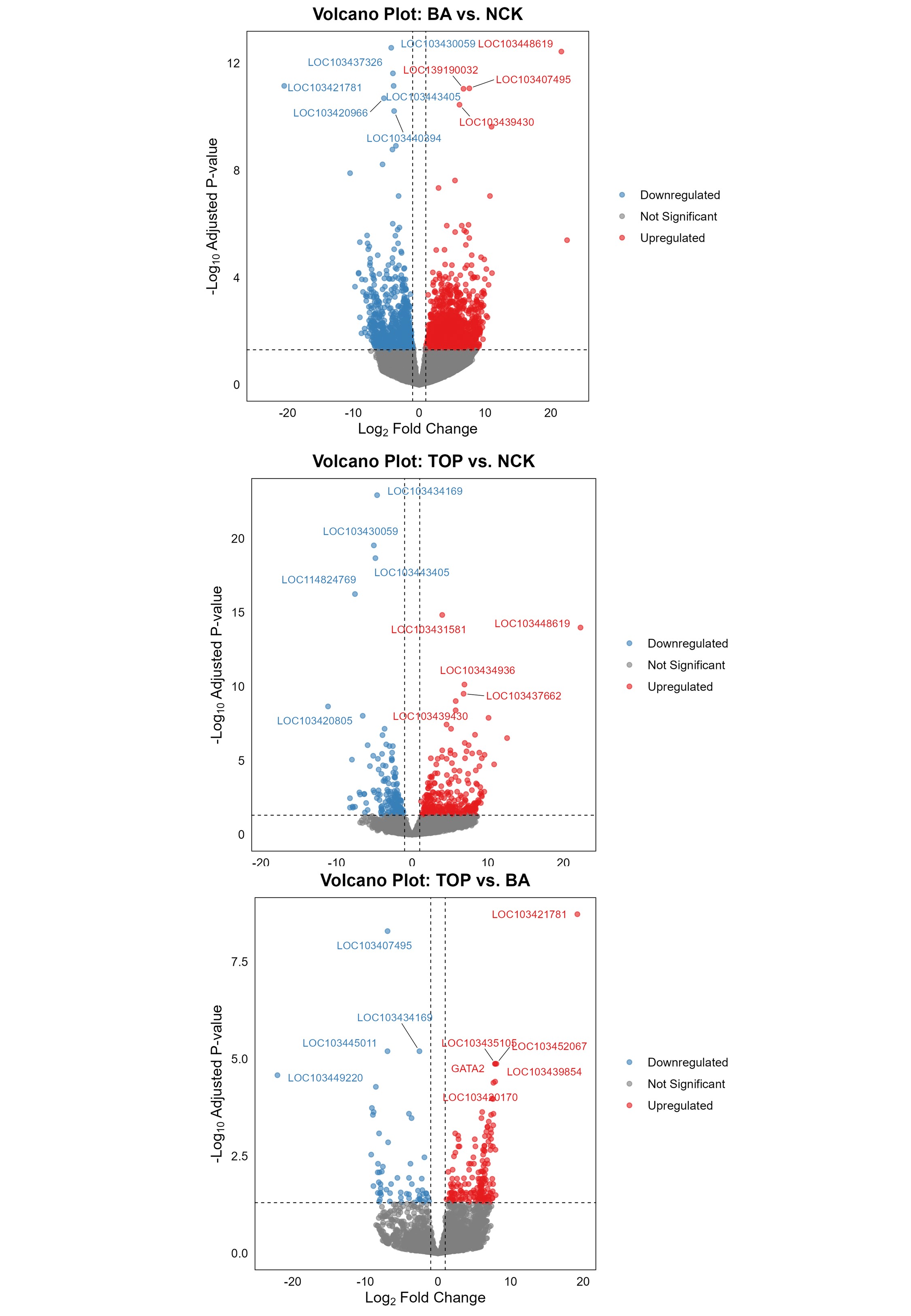

Supplement: Supplementary file 1 [file plants-14-03691-s001.zip › Figure S2.jpg]
